# Using Natural Language Processing to Construct a National Zoning and Land Use Database

**DOI:** 10.1177/00420980231156352

**Published:** 2023-03-16

**Authors:** Matthew Mleczko, Matthew Desmond

**Affiliations:** 1 Office of Population Research and School of Public and International Affairs; Princeton University, Princeton, NJ, USA; 2 Department of Sociology, Princeton University, Princeton, NJ, USA

**Keywords:** Zoning, housing, land use, natural language processing

## Abstract

In the United States, zoning and land use policies have been linked to high housing costs and residential segregation. Yet almost all zoning and land use data come from a handful of cross-sectional surveys, which are costly, time intensive, subject to low response rates and measurement error and are quickly dated. As an alternative, we construct the National Zoning and Land Use Database using natural language processing techniques on publicly available administrative data. We show this new database and our parsimonious measure of exclusionary zoning, the Zoning Restrictiveness Index, to be consistent with the Wharton Residential Land Use Regulatory Index (2018) and the National Longitudinal Land Use Survey (2019). Additionally, we overcome other limitations of these survey approaches, both by capturing previously omitted and important elements of land use policy and by revealing the land use regulations for a near-universe of municipalities in the San Francisco and Houston metropolitan statistical areas. We make all code and data publicly available, allowing the National Zoning and Land Use Database to be replicated in future years to ensure accurate, up-to-date and longitudinal nationwide zoning and land use data.

## Introduction

A considerable amount of empirical evidence indicates that restrictive zoning and land use policies, oftentimes referred to as exclusionary zoning, inflate housing prices and increase residential segregation (Glaeser and Gyourko, 2018; Rothwell and Massey, 2009; Sahn, 2022; Trounstine, 2018, 2020). Despite the clear importance of these policies, zoning and land use data, particularly of the longitudinal variety, are notoriously difficult to come by. The main sources of national zoning and land use data largely come from cross-sectional surveys. These surveys have been essential to documenting America’s zoning regime and to analyzing the role land use policies play in shaping communities, influencing housing costs and shoring up residential segregation (Hsieh and Moretti, 2019; Rothwell and Massey, 2009; Trounstine, 2020). However, researchers have noted that these surveys have their limitations (Manville, Lens, and Monkkonen, 2022; Rodríguez-Pose and Storper, 2020). In particular, we argue that they are costly, time intensive, subject to low response rates and measurement error and become dated soon after data collection.

As an efficient and accurate alternative to these survey methods, we access publicly available zoning and land use information and utilize natural language processing (NLP) to construct the National Zoning and Land Use Database (NZLUD). We begin by replicating selected measures using data collected from 2019–2022 for our target sample: the municipalities present in the 2006 Wharton Residential Land Use Regulatory Index (WRLURI) survey (Gyourko, Saiz and Summers, 2008). We discuss the process of downloading and parsing the zoning and land use text data and address how the NZLUD measures compare to the same measures among municipalities and metropolitan statistical areas (MSAs) present in the WRLURI 2018 database, a follow-up to the WRLURI 2006 survey. We also replicate measures of maximum permitted densities and inclusionary zoning programs from the 2003 National Longitudinal Land Use Survey (NLLUS), another land use database (Lo et al., 2019), comparing the NZLUD with estimates from the NLLUS 2019 database. Last, we combine all these measures to create the Zoning Restrictiveness Index (ZRI): a similar but distinct index compared to the WRLURI.

We show that municipalities and MSAs with the lowest and highest ZRI and WRLURI scores match reasonably well across the datasets. Moreover, sociodemographic characteristics of municipalities across the distribution of ZRI scores follow the same pattern established in previous studies (Gyourko, Saiz and Summers, 2008; Gyourko, Hartley and Krimmel, 2021). We also produce measures beyond previously existing zoning data, publishing what we believe to be the first national measures of accessory dwelling unit authorization, building height maximums, parking requirements and the proportion of residential districts permitting multi-family housing by right. These results suggest that utilizing municipal codes to construct a national zoning and land use database is a viable alternative to surveys. We make all code and data publicly available (see https://github.com/mtmleczko/nzlud), allowing the NZLUD to be replicated in future years to ensure accurate, up-to-date and longitudinal nationwide zoning and land use data.

### Zoning and land use data

Zoning and land use policies have critical implications for urban inequality and arguably represent the most significant power that local governments wield (Trounstine, 2018). Minimum lot sizes, use restrictions and height restrictions comprise three universal elements of zoning and land use policy, but many others exist (Fischel, 2015). The sheer number of ways municipalities can affect development presents profound challenges to measuring exclusionary zoning (Gyourko and Molloy, 2015).

The earliest versions of zoning laws sought to promote public health and sensible land use, but these policies were soon co-opted for exclusionary ends (Pendall, Puentes and Martin, 2006). Spearheaded by cities like San Francisco in the late 19^th^ century and Baltimore in the early 20^th^ century, municipalities began explicitly segregating by race in their zoning policies (Fischel, 2015; Nightingale, 2012). The Supreme Court case *Buchanan v. Warley* (1917) struck down the practice of racial zoning; however, enforcement was weak, and communities had other means to prevent integration, for instance, by implementing racial covenants (Fischel, 2015; Rothwell and Massey, 2009). Unlike racial zoning or covenants, exclusionary zoning (with race-neutral language) has survived many court challenges and remains widespread today (Fischel, 2015; Pendall, 2000).

A large body of empirical evidence has established a link between exclusionary zoning and higher housing prices, less housing construction and lower overall welfare (Hsieh and Moretti, 2019; Glaeser, Gyourko and Saks, 2005; Glaeser and Gyourko, 2018). Gyourko and Molloy (2015) provide a thorough review of this literature. An important and growing body of work also emphasizes the role of restrictive land use policies on increasing racial segregation (Pendall, 2000; Resseger, 2013; Rothwell and Massey, 2009; Rothwell, 2011; Sahn, 2022; Trounstine, 2020) and income segregation (Lens and Monkkonen, 2016; Rothwell and Massey, 2010).

As Gyourko and Molloy (2015) note, although researchers have established the centrality of zoning policies to a wide array of social problems and conditions, they lack access to national longitudinal zoning and land use data, which would help researchers identify the causal effects of exclusionary zoning (Quigley and Rosenthal, 2005). Some longitudinal zoning data do exist, but those typically only include a single state.^1^ The National Longitudinal Land Use Survey (NLLUS) is the most comprehensive national longitudinal data to date, but even these data cover only 88 MSAs between 2003 and 2019 and have even less coverage extending back to 1994 (Lo et al., 2019). Other longitudinal efforts have relied on state and federal land use and fair housing court cases to measure land use restrictiveness and fair housing injunctions (Ganong and Shoag, 2017; Trounstine, 2020). Finally, Gyourko, Hartley and Krimmel (2021) carried out the second wave of the WRLURI survey, which, when merged with the 2006 wave of the WRLURI survey produces data for 874 municipalities across two waves separated by thirteen years.

Despite these important efforts, data on zoning and land use still face two major hurdles: (1) the data by and large are not longitudinal and (2) they are not regularly updated. Moreover, most of these data are based on surveys, which are time-intensive, expensive and subject to low responses rates and measurement error. We overcome these limitations by constructing the National Zoning and Land Use Database (NZLUD) using natural language processing of municipal codes as well as zoning and land use ordinances.

### Constructing the database

To form our database, we replicated selected measures in the WRLURI 2006 and NLLUS 2003 data from municipal codes and zoning and land use ordinances collected between 2019 and 2022.^2^ We begin with a description of the WRLURI and NLLUS data as background into our data collection and processing procedure.

#### WRLURI data.

The WRLURI 2006 data represent the most widely cited and used land use data to date (Gyourko, Saiz and Summers, 2008). They cover a wide range of measures, from density restrictions and the timeliness of development approvals to the amount of local political pressure. The authors of these data condensed eleven subindex measures into one overall index of the land use regulatory environment: the WRLURI.

To build the WRLURI dataset, researchers mailed a survey questionnaire to 6,896 municipal officials between 2004 and 2006. The data contain 2,729 responses, yielding a response rate of roughly 40 percent, representing about 60 percent of the surveyed population. Larger municipalities were more likely to respond to the survey, but municipalities of varying sizes were well-represented in the data with the exception of towns with populations under 2,500.

In 2018, a team of researchers commissioned a new survey of land use regulations (Gyourko, Hartley and Krimmel, 2021). This survey mirrored the 2006 effort, asking many of the same questions during the 2018 calendar year. The 2018 update contains 2,844 responses, yielding a response rate of roughly 26 percent; 874 unique municipalities are represented in both datasets.

#### NLLUS data.

Compared to the WRLURI 2006 data, the NLLUS 2003 data cover less of the country but contain a more comprehensive set of zoning and land use measures (Gallagher, Lo and Pendall, 2019). The NLLUS 2003 data cover 36 states and Washington, D.C., encompassing 91 MSAs, while the WRLURI 2006 data cover 50 states and Washington, D.C., encompassing 323 MSAs. However, the NLLUS 2003 data tend to have better MSA-wide coverage, with a median percentage of municipalities represented of approximately 25 percent, versus roughly 12 percent in the WRLURI 2006 data.

The NLLUS 2003 data offer a complementary picture of the zoning and land use regulatory environment compared with the WRLURI 2006 data. They cover many of the same measures in addition to other important measures, such as the maximum permitted density in a municipality. The NLLUS 2003 survey generally targeted municipalities with populations over 10,000 in the 50 largest CMSAs (consolidated MSAs) and MSAs and produced a dataset of 1,845 jurisdictions, with a response rate of 78 percent. The NLLUS 2019 survey carried out a similar sampling procedure but produced data for only 1,703 jurisdictions and yielded a response rate of 58 percent (Lo et al., 2019).^3^

#### Survey limitations.

Fielding surveys, particularly national surveys, is expensive and time-consuming. This poses a challenge to any large-scale survey, but those of zoning and land use policies are especially susceptible to the inefficiency of survey data collection because zoning amendments take place regularly (Fischel, 2015). Consequently, some zoning data collected via survey could be inaccurate (or incomplete) by the time it is published.

Moreover, while survey efforts have yielded critical information, these surveys tend to have response rates below, and sometimes well below, 60 percent, lowering confidence in the data’s representativeness. Finally, land use surveys contain measurement error. Researchers commissioning zoning and land use surveys may receive duplicate responses with inconsistencies or different responses when multiple municipal officials submit survey responses. This is apparent in the WRLURI 2006 data, even for objective items such as minimum lot sizes. Accessing and parsing administrative source data offers more assurances against such inaccuracies.

#### Source data measures.

The WRLURI 2006 data provide a starting set of target municipalities and land use regulations. While our measures do not need to be consistent with the WRLURI measures, as the WRLURI data do not necessarily reflect the true underlying land use regulations in all cases, the WRLURI data do offer a useful benchmark to evaluate our measures. Consequently, we tailored our outputs and analyses to be consistent with the WRLURI 2006 and WRLURI 2019 studies, as well as the NLLUS 2003 and 2019 data.

We focused on a range of measures that were likely to be present in municipal zoning and land use regulations. We categorize these measures into the following five subindices:

1. Explicit growth controls index (EGCI): this subindex represents measures that directly restrict the supply of housing units and correlate with less housing density and displacement of low-income households and households of color (Levine, 1999). To keep consistent with WRLURI measures, we constructed this index as the sum of the six following growth controls, in the form of annual limits or caps on:
  - Single-family permits
  - Multi-family permits
  - Single-family units
  - Multi-family units
  - Multi-family dwellings
  - Multi-family dwelling units
2. Open space requirements index (OSRI): this subindex is a binary indicator of whether a municipality requires developers to dedicate or preserve some percentage of the lot(s) for open space or submit an in-lieu payment or fee, which can reduce the amount of land or funds available for development, particularly high-density development and affordable housing (Schmidt and Paulsen, 2009).
3. Minimum lot size index (MLSI): this subindex contains information regarding minimum lot sizes within municipal boundaries for all districts that allow residential uses. Larger minimum lot sizes restrict housing supply by implicitly restricting density (Fischel, 2015). In accordance with the WRLURI measures, we capture the maximum-minimum lot size across all residential districts, but we construct the MLSI as a categorical variable (rather than a binary indicator of the top two minimum lot sizes), which could fall into one of the following mutually exclusive categories:
  - Less than one-half acre
  - Greater than or equal to one-half acre and less than one acre
  - Greater than or equal to one acre and less than two acres
  - Greater than or equal to two acres
4. Reviewing and/or approving authorities for projects not requiring rezoning (NZI): this subindex represents the sum of the following five governing bodies needed to approve a project that does not require rezoning:
  - Planning commission
  - Local municipal council
  - County board
  - Environmental review board
  - Public health board
  - Design review board
5. Reviewing and/or approving authorities for rezoning (RZI): this subindex represents the sum of how many of the following governing bodies need to approve rezonings (including variances) or zoning amendments:
  - Planning commission
  - Local municipal council
  - Zoning board
  - County board
  - County zoning authority
  - Environmental review board
  - Town meeting
  We also replicated two pre-existing measures contained in the NLLUS 2003 data:
6. Inclusionary zoning programs index (IZPI): this subindex is a binary indicator for municipalities that operate inclusionary zoning programs, including the use of in-lieu payments or fees often directed towards affordable housing trust funds (Pendall, Puentes and Martin, 2006).^4^
7. Maximum permitted density index (MPDI): this subindex indicates the maximum permitted number of dwelling units per acre (or square feet of lot area per acre) in any district that allows residential uses. It features the following categories, expressed in units per acre:
  - 0–4
  - 5–7
  - 8–14
  - 15 to 30
  - More than 30
  Our data contain a subset of measures included in the overall WRLURI. While our data capture a narrower scope of land use regulations, they also include more information regarding inclusionary zoning. In particular, we observe maximum permitted density, identified as a variable of key importance in prior work on zoning and residential segregation (Rothwell and Massey, 2009, 2010).
  Moreover, in addition to the regulations captured in the WRLURI and NLLUS data, we demonstrate the scalability of our approach by calculating four additional and important measures of zoning and land use policy:
8. Maximum height index (MHI): recognizing building height restrictions as a key method for restricting density (Brueckner and Singh, 2020), we collect the median and mode building height limits (in feet and stories) across all districts in which residential uses are permitted, taking the median of these four numbers.^5^
9. Minimum required parking index (MRPI): to capture another common mechanism for limiting density (Bronin and Ilyankou, 2021), we compute the median and mode of parking requirements per residential unit in districts permitting residential uses and take the average of these two numbers.
10. Accessory dwelling unit index (ADUI): this subindex is a binary measure that captures whether or not a municipality permits accessory dwelling unit (ADU) construction in any residential district at all.^6^ ADUs are increasingly seen as a way to promote moderate housing density and lower housing construction costs (Brinig and Garnett, 2013).
11. Permitted multi-family housing index (MFPI): we constructed this subindex, the proportion of residential districts that allow multi-family housing development by right, as a way to capture a municipality’s preference for single-family zoning, one of the most recognized and explicit forms of exclusionary zoning (Sahn, 2022).^7^

#### Natural language processing.

Zoning and land use regulations are ubiquitous and often available online. In fact, over 95 percent of the municipalities represented in the WRLURI 2006 sample have made their zoning and land use information publicly available online.

We began with these 2,660 municipalities (out of the 2,720 unique municipalities in the 2006 WRLURI sample).^8^ When possible, we downloaded the entirety of the municipal codes to ensure that we captured all information pertinent to zoning and land use.^9^ Despite many idiosyncrasies, most zoning and land use bylaws contain sections pertaining to district use restrictions, dimensional requirements and administrative processes, which is where the bulk of our extracted information was located. Ultimately, we downloaded and verified 2,639 sets of municipal codes, representing about 97 percent of the original target sample.^10^ The other 3 percent of municipalities do not make their codes publicly available.

After verifying that the raw data contained pertinent zoning and land use information, we then utilized natural language processing (NLP) to transform the text data into a usable file for analysis. Our pre-processing steps included lemmatization of keywords, the filtering of unnecessary words, punctuation marks, or symbols and standardizing numeric information (either stored as words or fractions) into digits. To circumvent the lack of standardization in the text, we built a series of regular expression searches that matched on a set of original keywords for each of our target measures, capturing a specified number of preceding and following characters depending on the measure.^11^ Through a process of trial-and-error, we also defined a range of words or phrases that, when present in a given text string, indicated a false positive match and prevented any further matching for the given text string.

In the text that follows, we describe our general text processing procedure. We provide specific details about the construction of each measure in the Supplementary Material.

For our non-dimensional measures (EGCI, OSRI, NZI, RZI, IZPI and ADUI indices), we searched for additional keywords within a captured text string. Each of these keywords had an associated weight. We summed these weights of the matching keywords within a captured text string. When the sum of the weights was greater than the established threshold, we set the indicator column for a particular measure to 1. Otherwise, we set the indicator column to 0. See Supplementary Material Table S2 for a list of keywords used and their associated weights.

The challenge with this approach consisted of limiting false-positive matches (by filtering inappropriate matches) and minimizing false-negative non-matches (by capturing as many potential matches as possible) for the above measures. For instance, inclusionary zoning programs have many names (e.g., inclusionary housing, incentive housing, density bonuses) and oftentimes entail different incentives. Validating this step required extensive quality checks to ensure that our set of matching words and stop-word and stop-phrase filters appropriately captured the breadth of key terms while also screening out inappropriate matches.

Our dimensional measures (MLSI, MPDI, MHI, MRPI, and MFPI indices) required a different process. Underlying information for these measures largely came from in-text district regulations, tables of dimensional requirements, or both (see Figures S1 and S2 in the Supplementary Material for illustrations). In the case of the in-text references, our code searched within the original captured string, advancing to smaller strings of text surrounding the original keyword by passing stop-phrase flags and matching key contextual phrases to extract the relevant information. In the case of information stored in dimensional requirement tables, we built our code to detect the presence of these tables and extract the relevant dimensional requirements from them.

Verifying this process required overcoming a series of complications. For one, we accounted for information stored in various formats, such as minimum lot sizes stored in square feet or acres, maximum permitted densities stored as units per acre or square feet per acre and building height maximums stored in feet or stories. Additionally, to compute a per-unit minimum lot size metric, this approach required us to restrict our matches to the relevant portions of residential district requirements. This entailed not only filtering out the same measures for non-residential districts (e.g., commercial, industrial), but also some dimensional requirements relevant to multi-family housing developments, planned unit developments (PUDs), cluster developments, or subdivisions, which often express requirements in terms of the entire development rather than each individual lot. Similarly, we filter out dimensional requirements such as floor space requirements and minimum unit sizes, which capture regulations that are similar but distinct from minimum lot sizes. We accomplished this through a series of checks for stop-phrases and filtering out of implausible dimensional requirement matches.^12^

This process outputs all captured minimum lot sizes, maximum permitted densities, maximum building heights and per-unit parking requirements across all residential districts or instances in which residential units are permitted, whether by right or through conditional or special use. This latter condition captures all residential development that could be undertaken, including rural, overlay, or agricultural districts where large-lot single-family dwellings are permitted, capturing an important dimension through which municipalities could limit density (Fischel, 2015).^13^

We calculate the proportion of districts permitting multi-family housing by right among residential and mixed-use districts by assigning each identified and eligible district a specific permitted use category for each mention of its by right permitted uses in the text. The permitted use categories include single-family, multi-family (i.e., four-family units or more), two-family (including attached single-family units, townhomes and triplexes) and mixed-use.^14^ We assign each eligible district to a permitted use category based on its modal permitted use assignment. The sum of districts assigned to multi-family or mixed uses by right form the numerator of our measure, while the total number of assigned districts forms the denominator. See the Supplementary Material for more details.

### Post-processing.

The resulting output of all text processing contained 33 binary indicators and 7 numeric indicators, corresponding to the previously discussed measures.^15^ We iterated this process, checking the aggregate means across our measures for face validity in addition to systematically checking the validity of values in the highest categories (e.g., maximum minimum lot sizes of 2 or more acres). In the case of minimum lot size and maximum permitted density measures, we checked output values against the municipal codes among roughly a quarter of our sample to verify their accuracy.^16^

We used the minimum lot size information collected to impute the maximum permitted density for two municipalities. Our code failed to capture minimum lot size information for about 4 percent of the sample. In these cases, we manually inputted the minimum lot size information. Additionally, a quarter of the sample required manual formatting edits to the municipal codes to produce accurate minimum lot size and maximum permitted density measures.^17^

To condense the information conveyed in these indicators, we created an index similar to the WRLURI but with slightly different measures. As in Gyourko, Saiz and Summers (2008), we employ principal components analysis of our eleven subindices to create our overall standardized index, which we label the Zoning Restrictiveness Index (ZRI). We extract the first principal component and create the ZRI (prior to standardizing) as a weighted linear combination of the subindex values, with the resulting loadings serving as the weights. Additionally, to compare our output against the WRLURI output, we re-create the ZRI using only measures that exist in both our data and the WRLURI and NLLUS data.^18^See Supplementary Material Table S3 for more details about the construction of the ZRI and its subindices.

To benchmark our results, we also create parallel ZRI indices, this time using values from the WRLURI and NLLUS data sources. We were able to merge 874 municipalities represented in both waves of the WRLURI survey, 591 municipalities in the 2019 NLLUS survey and 242 municipalities in both the NLLUS 2019 and the two WRLURI survey waves.^19^ This resulted in three subsamples of municipalities with which to compare the results of our data creation. Additionally, as with both WRLURI studies, we specify survey weights to account for potential bias from missing data. Details on the construction of these weights along with ZRI summary statistics when applying them are contained in Supplementary Tables S5 and S6, respectively.

We also aggregated municipalities to the MSA level and computed several unweighted MSA-level ZRI indices. These included the median ZRI across municipalities present in the data for a given MSA, the difference between the highest suburban ZRI (i.e., non-central city) and central city ZRI, and a standardized combination of the two. Similar to prior studies (Trounstine, 2018), we use this combined index as our primary MSA-level ZRI since it captures the average restrictiveness across an MSA, but also the extent to which there exist highly exclusive suburban municipalities relative to central city municipalities. We compare this measure to the standardized median of 2018 WRLURI scores for each MSA.

These comparisons allow us to gauge the consistency of our data with the two other established sources of national zoning and land use information. While a high degree of consistency is desirable, capturing the zoning and land use regulations accurately is more important. Here, our method improves upon previous methods as we compare our results against the actual regulations, particularly when values differ across datasets. A correlation coefficient of roughly 0.3 between the ZRI and WRLURI indicates that while both indices generally measure a similar concept, they are nonetheless different measures of zoning restrictiveness.

## Results

Despite the complexity of building a zoning and land use database from administrative text data, we constructed a database with values comparable to the WRLURI 2018 and NLLUS 2019 data. Tables 1 and 2 display the results. We achieve similarity in aggregate means across both full samples for each indicator. Our data record higher fractions of municipalities with minimum lot sizes of one or two acres or more as well as higher permitted densities.^20^ Nevertheless, the aggregate means for each minimum lot size and maximum permitted density indicator point to generally similar levels of permitted density across both datasets.

To assess municipality-specific congruence across datasets, we turn to municipal-level comparisons for the 874 matching WRLURI municipalities and 591 matching NLLUS municipalities, captured by the final three columns of Tables 1 and 2, respectively. Again, we note the similarities in mean values across the matching datasets and consistency with the trends in mean values across the full datasets. Moreover, the fraction of matching values across the datasets is large for most of the measures.

The measures with the lowest fraction of matching values are those most difficult to measure. For instance, most municipalities have open space regulations of some kind. Distinguishing between these and whether a municipality requires “mandatory dedication of space or open space (or fee in lieu of dedication)” likely has led to measurement error in survey data (Gyourko, Hartley and Krimmel, 2021: Online Appendices p. 5). Additionally, while we achieve notable matching rates for several individual reviewing or approving authority measures (see Table S7 in the Supplementary Material), distinguishing between approval authority and responsibility to review and perhaps provide recommendations also has likely led to measurement error in previous data.

Tables 3 and 4 respectively display the ten most and least restrictive MSAs, with at least ten municipalities present in the data according to the ZRI and WRLURI. Table 3 illustrates that the MSAs our method identifies are consistent with prior research and reflect many previously identified in the literature as highly restrictive and suffering from acute housing shortages (Gyourko, Hartley and Krimmel, 2021). Notably, our method identifies the Washington, D.C. MSA as the most restrictive MSA, followed by the New York City MSA, the most restrictive MSA according to the WRLURI. While these results suggest considerable overlap between the ZRI and WRLURI, there are still some notable differences. In particular, we identify the Detroit MSA, one of the least restrictive MSAs according to the WRLURI results, as the eighth most restrictive in our data. This result serves as a reminder that our multi-dimensional MSA-level ZRI is a measure of exclusionary zoning that is distinct from the WRLURI. In addition to capturing average MSA-wide zoning and land use restrictiveness (similar to the WRLURI), our MSA-level ZRI reflects divergent zoning and land use restrictiveness between a central city and suburban municipalities as reflected in the Detroit MSA.

The regions present in Table 4 differ from those in Table 3, with a more pronounced presence of MSAs from the South and Midwest regions. Nevertheless, there is regional overlap across the samples in Table 4, further supporting the conclusion that both methods are broadly similar in their characterization of land use restrictiveness. Moreover, consistent with Gyourko, Saiz and Summers (2008) and Gyourko, Hartley and Krimmel (2021), we assess the sociodemographic profile of municipalities along quantiles of ZRI scores, finding that zoning restrictiveness, on average, tends to be higher in more affluent, diverse and dense municipalities. The same is true for MSAs, though our results demonstrate that more restrictive MSAs also tend to be more segregated. Table S8 in the Supplementary Material contains these results.

### Overcoming the limitations of prior zoning data

Having validated our measures with previously existing zoning and land use data, we turn now to demonstrating the extent to which our method can be scaled to include additional measures and additional municipalities. First, we consider the additional measures that we created beyond those found in the WRLURI or NLLUS data (see Table S9 in the Supplementary Material). Roughly 46 percent of municipalities in the sample permit some form of accessory dwelling unit (ADU). The average maximum building height across the sample registers at 34 feet and the average required number of parking spaces per residential unit is 1.8. Finally, on average, nearly 39 percent of residential districts in a municipality permit multi-family housing development by right, though this varies considerably across the sample, as 253 municipalities (approximately 10 percent of the sample) do not permit any multi-family housing by right in any residential district.

A major disadvantage of previously available national zoning data is under-coverage within MSAs. For instance, the median proportion of municipalities in a given MSA represented in the WRLURI data is 0.12. Since our approach can create zoning data for any municipality for which there is publicly available text data, we can scale our approach by expanding our sample. As an illustration, we download all publicly available municipal codes for two often-referenced MSAs in the zoning literature: San Francisco and Houston (Gyourko, Hartley and Krimmel, 2021). This allows us to assess how sensitive our measures are to within-MSA under-coverage and to generate a more complete picture of within-MSA zoning regimes across MSAs.

We attained 100 percent coverage in the San Francisco MSA and 70 percent coverage in the Houston MSA (as opposed to 38 and 16 percent, respectively, in the WRLURI sample). While the coverage in the Houston MSA is notably lower, the municipalities with missing zoning text data tended to be very small and sparsely populated. The municipalities for which we do have coverage represent 99% of the Houston MSA population and 95% of its land area.

**Figure 1.**
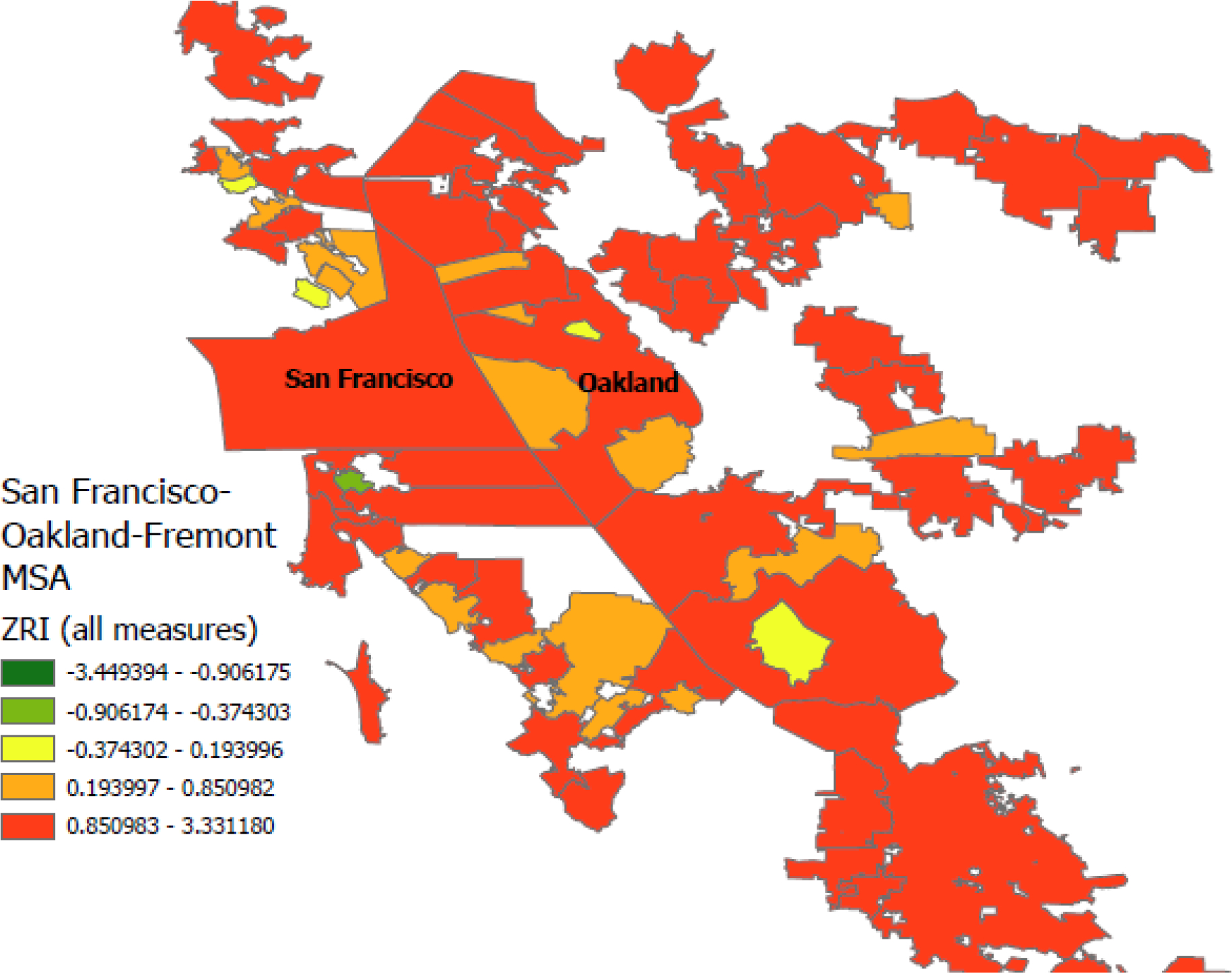
ZRI (all measures) values for the complete San-Francisco-Oakland-Fremont MSA

**Figure 2.**
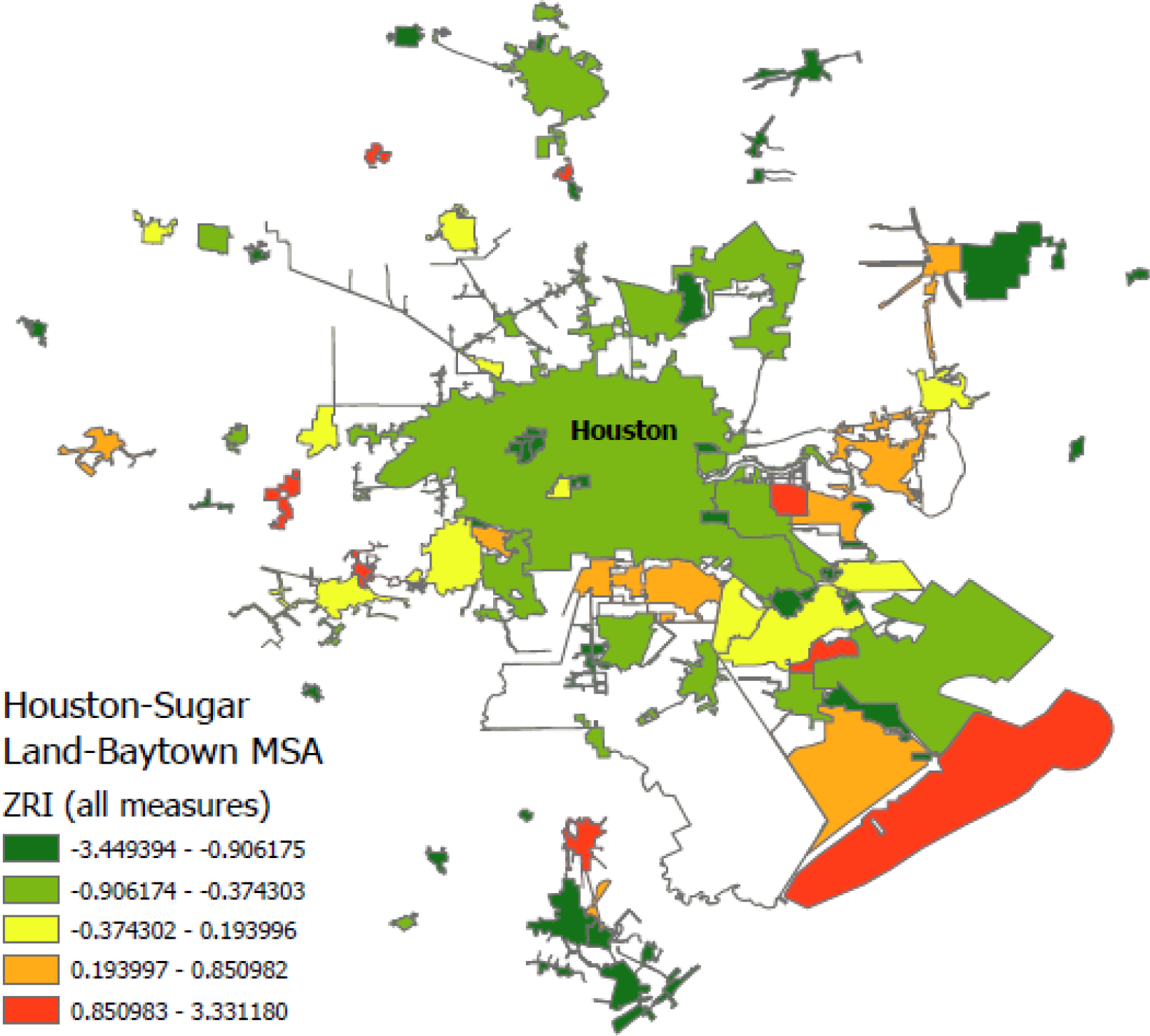
ZRI (all measures) values for the complete Houston-Sugar Land-Baytown MSA

Figures 1 and 2 display maps with graduated color scales indicating quintiles of the ZRI with all eleven subindices for municipalities within the San Francisco and Houston MSAs, respectively. One can immediately see that average zoning restrictiveness is higher in the San Francisco MSA. Nearly every municipality in the San Francisco MSA registers ZRI scores in the fourth or fifth quintile. Alternatively, many municipalities in the Houston MSA are characterized by relatively permissive zoning and land use regulations. A number of municipalities, including the central city Houston, have no zoning codes at all, but still possesses policies governing land use to some degree. Additionally, while median zoning restrictiveness in the Houston MSA is lower than in the San Francisco MSA, there are still examples of exclusionary suburbs within the Houston MSA (e.g., Fulshear), demonstrating that some aggregate MSA measures of zoning restrictiveness can mask within-MSA exclusionary dynamics, which further motivates the use of our multi-dimensional MSA-level ZRI index.

Finally, by collecting the total or near-universe of municipalities within an MSA, we can assess the robustness of our ZRI measures to excluding potentially significant portions of an MSA or elements of land use policy (see Table S10 in the Supplementary Material). We find that adding measures of land use policy generally attenuates the ZRI results but including full sample coverage accentuates them. Neither addition substantially altered the direction or general magnitude of the results when compared to ZRI constructions with fewer measures or incomplete sample coverage. These results indicate that incorporating additional data into our text-processing can produce more precise and complete zoning and land use data, while still affirming that previous MSA-level results using a subset of these measures or samples can still be broadly consistent with these new data.

## Discussion

Applying natural language processing to administrative data from across the country, we created the National Zoning and Land Use Database (NZLUD), finding that it compares well to survey data that have been the most frequent form of zoning and land use measurement up until this point. We have shown our process to be viable and accurate for the most readily identifiable forms of land use regulation in municipal codes. Our efforts, when combined with pre-existing and publicly available zoning data, can create a longitudinal dataset of zoning and land use policies using a process that is cost-efficient, replicable, accurate and less prone to measurement error, overcoming many of the challenges endemic to survey studies of this topic. Because we have made our data and code public, future iterations of these data can expand to other measures and municipalities not captured in the WRLURI or NLLUS samples. This process can be refreshed at regular intervals to provide consistent and accurate panel data of zoning and land use policies from across the United States.

Our contribution is notable for its use of NLP techniques to uncover zoning and land use policies across the country. It also complements similar efforts, including both statewide and national studies that have manually processed zoning ordinances and maps (Sahn, 2022; Bronin and Ilyankou, 2021). Similarly, Shanks (2021) uses NLP techniques to construct an index of zoning restrictiveness – the Natural Language Processing Zoning Stringency Index (NALPZ) – from municipal zoning bylaws in Massachusetts. The Urban Institute has utilized machine learning techniques to predict permitted densities from geospatial zoning data and zoning ordinances (Tyagi and MacDonald, 2019). Finally, Song (2021) estimates minimum lot sizes for a near-universe of single-family zoning districts across the U.S. via a structural-break algorithm using parcel and HMDA data. Notwithstanding some differences, our approach generally leads to similar conclusions from these efforts regarding the pervasiveness of restrictive zoning and land use.^21^

However, our approach stands out from these efforts for a number of reasons. First, our method produces a dataset with dozens of measures, as opposed to a single measure (Song, 2021; Shanks, 2021), which is important given how no single dimension of zoning and land use regulations can capture the full extent of zoning restrictiveness. Second, because our data can be checked against publicly available information, our method is falsifiable, which distinguishes it from approaches like Shanks (2021). Third, our data provides greater coverage, both geographically (by including more than just one state) and topically (our data applies to all residential development, not just single-family zoned properties). Finally, we provide the source code that can be adapted and supplemented, representing the most open-source and automated process of collecting nationwide zoning and land use data to date.

Our approach, despite its promise, is not without its limitations. First, while our approach is less time-intensive and more scalable compared to survey methods, collecting the input zoning and land use text information still requires a considerable amount of time and attention to ensure that all relevant zoning and land use information are present in the text corpus. Second, a non-trivial portion of the sample required manual formatting overrides to produce accurate information for certain measures, though future refinements to the code should be able to limit the need for this moving forward. Finally, our approach misses other important elements of zoning and land use information, namely, data stored in zoning maps that reveal how much land a municipality zones for multi-family housing (Ellickson, 2021). Incorporating zoning map data into our procedure could be a worthwhile avenue for future research.

Researchers and advocates should continue to investigate zoning and land use policies, not only owing to their role in inflating housing prices and preserving residential segregation but also as an avenue for possible reform. To assess and identify the disparate impact of zoning and land use policies and to actively monitor policy outcomes, researchers need more complete and timely access to data. Our contribution represents an important step forward in this regard.

## Supplementary Material

Online appendix

## Figures and Tables

**Table 1.** Comparison of National Zoning and Land Use Database output to Wharton Residential Land Use Regulatory Index 2018 output

Measure	All municipalities		Municipalities in 2004–2006 and 2018 Wharton Residential Land Use Regulatory Index samples		
	National Zoning and Land Use Database (N=2,639)	Wharton Residential Land Use Regulatory Index 2018 sample (N=2,844)	National Zoning and Land Use Database (N=874)	Wharton Residential Land Use Regulatory Index 2018 sample (N=874)	Matching values (N=874)
Restrict single-family permits	0.02	0.02	0.02	0.02	0.97
Restrict multi-family permits	0.02	0.03	0.02	0.03	0.96
Limit single-family units	0.03	0.02	0.03	0.02	0.96
Limit multi-family units	0.03	0.03	0.03	0.03	0.95
Limit multi-family dwellings	0.02	0.03	0.02	0.03	0.96
Limit multi-family dwelling units	0.02	0.04	0.02	0.03	0.96
Any minimum lot size	0.99	0.91	1.0	0.96	0.96
Minimum lot size: less than 0.5 acre	0.43	0.38	0.40	0.50	0.67
Minimum lot size: between 0.5 acre and 1 acre	0.14	0.15	0.14	0.17	0.79
Minimum lot size: between 1 acre and 2 acres	0.18	0.12	0.20	0.12	0.78
Minimum lot size: 2 acres or more	0.25	0.24	0.26	0.22	0.75
Open space	0.68	0.54	0.70	0.59	0.60
Mean number of reviewing/approving authorities: no rezoning	2.0	1.5	2.1	1.5	0.38
Mean number of reviewing/approving authorities: rezoning	2.7	2.8	2.7	2.8	0.35

Note: Minimum lot size categories are coded to be mutually exclusive. Some proportions may not sum to 1 because of missing values.

**Table 2.** Comparison of National Zoning and Land Use Database output to National Longitudinal Land Use Survey 2019 output

Measure	All municipalities		Municipalities in National Zoning and Land Use Database and National Longitudinal Land Use Survey 2019		
	National Zoning and Land Use Database (N=2,639)	National Longitudinal Land Use Survey 2019 (N=1,443)	National Zoning and Land Use Database (N=591)	National Longitudinal Land Use Survey 2019 (N=591)	Matching values (N=591)
Inclusionary zoning/any affordable housing program	0.29	0.38	0.42	0.42	0.81
Maximum permitted density: 0–4 dwelling units/acre	0.04	0.18	0.02	0.09	0.82
Maximum permitted density: 5–7 dwelling units/acre	0.05	0.14	0.03	0.14	0.76
Maximum permitted density: 8–14 dwelling units/acre	0.14	0.21	0.14	0.20	0.68
Maximum permitted density: 15–30 dwelling units/acre	0.31	0.20	0.24	0.23	0.61
Maximum permitted density: 31 or more dwelling units/acre	0.47	0.27	0.56	0.34	0.56

Note: Maximum permitted density categories are coded to be mutually exclusive. Some proportions may not sum to 1 because of missing values.

**Table 3.** Comparison of most restrictive MSAs between National Zoning and Land Use Database and Wharton Residential Land Use Regulatory Index 2018 sample

Rank	MSAs with at least 10 responses					
	National Zoning and Land Use Database (N=48)			Wharton Residential Land Use Regulatory Index 2018 sample (N=54)		
	MSA	Municipalities in data	Zoning Restrictiveness Index	MSA	Municipalities in data	Wharton Residential Land Use Regulatory Index
1	Washington-Arlington-Alexandria, DC-VA-MD-WV	15	2.05	New York-Northern New Jersey-Long Island, NY-NJ-PA	51	1.74
2	New York-Northern New Jersey-Long Island, NY-NJ-PA	108	2.01	San Francisco-Oakland-Fremont, CA	20	1.73
3	Providence-New Bedford-Fall River, RI-MA	21	1.81	Phoenix-Mesa-Glendale, AZ	11	1.24
4	Seattle-Tacoma-Bellevue, WA	27	1.60	Providence-New Bedford-Fall River, RI-MA	14	1.22
5	Tampa-St. Petersburg-Clearwater, FL	12	1.44	Riverside-San Bernardino-Ontario, CA	18	1.14
6	Milwaukee-Waukesha-West Allis, WI	23	1.27	Seattle-Tacoma-Bellevue, WA	22	1.07
7	Miami-Fort Lauderdale-Pompano Beach, FL	36	1.20	Portland-Vancouver-Hillsboro, OR-WA	19	1.00
8	Detroit-Warren-Livonia, MI	50	1.14	Miami-Fort Lauderdale-Pompano Beach, FL	39	0.92
9	Boston-Cambridge-Quincy, MA-NH	57	1.14	Washington-Arlington-Alexandria, DC-VA-MD-WV	18	0.82
10	Springfield, MA	16	1.01	Philadelphia-Camden-Wilmington, PA-NJ-DE-MD	59	0.81

**Table 4.** Comparison of least restrictive MSAs between National Zoning and Land Use Database and Wharton Residential Land Use Regulatory Index 2018 sample

Rank	MSAs with at least 10 responses					
	National Zoning and Land Use Database (N=48)			Wharton Residential Land Use Regulatory Index 2018 (N=54)		
	MSA	Municipalities in data	Zoning Restrictiveness Index	MSA	Municipalities in data	Wharton Residential Land Use Regulatory Index
1	San Antonio-New Braunfels, TX	13	−1.44	Lansing-East Lansing, MI	11	−0.74
2	Oklahoma City, OK	13	−1.05	Akron, OH	11	−0.71
3	Columbus, OH	11	−0.39	St. Louis, MO-IL	41	−0.56
4	San Diego-Carlsbad-San Marcos, CA	11	−0.15	Detroit-Warren-Livonia, MI	69	−0.55
5	Pittsburgh, PA	44	−0.04	Dayton, OH	13	−0.55
6	Des Moines-West Des Moines, IA	11	0.01	Grand Rapids-Wyoming, MI	19	−0.53
7	Rochester, NY	12	0.12	Rochester, NY	23	−0.51
8	Denver-Aurora-Broomfield, CO	12	0.12	Louisville/Jefferson County, KY-IN	11	−0.45
9	Manchester-Nashua, NH	11	0.18	Cincinnati-Middletown, OH-KY-IN	35	−0.42
10	Atlanta-Sandy Springs-Marietta, GA	30	0.23	Kalamazoo-Portage, MI	11	−0.38

## Data Availability

Repository: https://github.com/mtmleczko/nzlud Data are available under the MIT License.

## Footnotes

1 See Massachusetts (Glaeser, Schuetz and Ward, 2006) and California (Glickfeld et al. 1999; Lewis and Neiman 2000; Mawhorter and Reid, 2018).

2 We included in the output data the timestamp of when the municipal codes were downloaded or reformatted.

3 The NLLUS 2019 ultimately contains 1,530 municipalities, as 173 of these jurisdictions are county governments.

4 The WRLURI 2006 question measuring inclusionary zoning seems to solely capture mandatory inclusionary zoning programs, which possibly misses many incentive-based inclusionary zoning programs. For this reason, we use the more comprehensive NLLUS measure of inclusionary zoning.

5 We convert building stories to feet by defining one story as 14 feet.

6 ADUs go by many names (e.g., granny flats, accessory apartments, guest homes), so we include a myriad of matching words to capture any mention of these regulations.

7 This measure is distinct from the proportion of a municipality’s land zoned for multi-family housing advocated for elsewhere (Ellickson, 2021). However, this measure still captures a dimension of proclivity to single-family zoning and can identify jurisdictions that do not permit multi-family housing by right in any district.

8 Of the 2,729 observations in the WRLURI 2006 dataset, we drop two municipalities that were later annexed and three duplicated municipalities with missing information. We take the average across values for four duplicated municipalities.

9 The contents of these codes are oftentimes stored in external PDF files and lack standardization, even within the same vendor, which renders web-scraping ineffectual. If a municipality stored its zoning and land use information in the form of scanned PDFs (about 5 percent of the sample), we made use of optical character recognition technology in Adobe Acrobat Pro to convert the scanned PDF into text.

10 We applied the county zoning and land use policies to a handful of municipalities located in counties that have primary zoning and land use authority.

11 For dimensional regulation tables or use restrictions, we captured the preceding 300 and following 2,000 characters. For parking measures, we captured the preceding and following 750 characters. For all other measures, we captured the preceding and following 250 characters.

12 We filter out the following dimensional requirement matches: acres pertaining to agricultural districts over 50, acres for all other districts over 5, units per acre over 165, square feet per unit under 380, building heights under 10 feet, and parking spaces per unit over 10.

13 If our code detects that the matched string pertains to a district that does not permit residential uses whatsoever, we do not extract any information from the matched string.

14 We generally did not use information from tables of permitted uses because of how widely their formats varied. Additionally, when a district recorded multiple matches for a particular string and triggered the appropriate flag, we assigned a match based on the following hierarchy: any multi-family match indicates permitted multi-family use, followed by mixed-use, followed by two-family, followed by single-family.

15 We were unable to convert the codes into a text file for one municipality, in which case we manually input the correct values for each measure.

16 We generally constructed this subset from municipalities that did not have matching values for minimum lot size and maximum permitted density indicators. We did the same for other measures like open space requirements and inclusionary zoning programs, but explicitly checked these values among smaller subsets (5–6 percent of the sample).

17 These instances involved minor, but manual formatting fixes that our code could not accommodate. For instance, when downloading codes from some vendors (e.g., ecode360), oftentimes the location of headings and subheadings moved, sometimes in ways that would trigger a stop phrase or a false-positive match. Additionally, important table formatting was oftentimes lost in the data downloads, stored in a complex format, or subject to formatting glitches (e.g., a footnote is incorrectly converted into an additional digit), making it difficult for our code to correctly parse dimensional regulation tables.

18 This first principal component explains just under 23 and 17 percent of the overall variance for the reduced and full ZRIs, respectively.

19 We utilized crosswalks from the U.S. Census Bureau in order to standardize geoids across all datasets. These include the 2019 State, County, Minor Civil Division and Incorporated Place FIPS Codes file, the March 2020 CBSA delineation file, 2010 place/county subdivision level FIPS code files and place/county to CBSA crosswalks from the Missouri Census Data Center.

20 One reason for our higher fraction of two-acre minimum lot sizes is the number of large-lot, agricultural zoned districts that allow for single-family detached residences in addition to agricultural activities. Our data reflect these minimum lot sizes while the WRLURI data, in many cases, do not.

21 For instance, Shanks (2021) estimates the highest levels of zoning and land use restrictiveness in rural parts of Massachusetts as opposed to major metropolitan areas such as Boston, where our method estimates the highest levels of restrictiveness in the state.
