# Supplementary material for: Using Natural Language Processing to Construct a National Zoning and Land Use Database
Source: Urban Stud. Author manuscript; Available in PMC 2025 Jan 16. (PMC11737588; doi:10.1177/00420980231156352)
Supplement: Online appendix [file NIHMS1925852-supplement-Online_appendix.pdf]

Listing of Supplemental Material contents

- Table S1 lists the municipal code vendors and repositories that we used to access municipal codes outside of municipal websites.
- Table S2 details the matching words and weights used to construct the measures in our data. We also provide additional details about the process for particular measure constructions.
- Table S3 includes information about the principal components analysis, including the ZRI loadings.
- Table S4 ranks the top-10 average values for a selected number of ZRI subindices.
- Table S5 contains results for the logistic regressions used to estimate survey weights.
- Table S6 details summary statistics for the ZRI with and without applying stabilized sample weights.
- Table S7 details the comparison of NZI and RZI components against the WRLURI 2018 sample.
- Table S8 details the sociodemographic profile of municipalities and MSAs for quantiles of ZRI scores.
- Table S9 displays the means for ADU authorization, maximum permitted building heights, minimum parking requirements and proportions of residential districts permitting multifamily housing by right.
- Table S10 contains the results for ZRI constructions using additional subindices and/or additional municipalities for the San Francisco and Houston MSAs.
- Table S11 contains a comparison of the most restrictive municipalities between the full NZLUD and WRLURI 2018 samples.
- Table S12 contains a comparison of the most restrictive municipalities among municipalities in the NZLUD, WRLURI 2018 and NLLUS 2019 samples.
- Table S13 details the comparison of the most restrictive MSAs between the full NZLUD and WRLURI 2018 samples.
- Table S14 contains a comparison of the least restrictive municipalities between full NZLUD and WRLURI 2018 samples.
- Table S15 details the comparison of the least restrictive municipalities among municipalities in NZLUD, WRLURI 2018 and NLLUS 2019 samples.
- Table S16 contains the comparison of the least restrictive MSAs between full NZLUD and WRLURI 2018 samples.
- Figure S1 provides an example of dimensional requirements stored in text.
- Figure S2 provides an example of dimensional requirements stored in a table.
- Figure S3 details the comparison of states with the most inclusionary zoning programs between the NZLUD and NLLUS 2019 data.
- Figure S4 illustrates density plots of ZRI (NZLUD) and WRLURI (WRLURI 2018) indices among all municipalities.
- Figure S5 illustrates density plots of ZRI (NZLUD) and WRLURI (WRLURI 2018) indices among all MSAs.

Table S1. Listing of municipal code vendors or repositories utilized

**Vendor/repository**
Municode
American Legal Publishing Company
Code Publishing, Inc
Sterling Codifiers
General Code (eCode360)
Franklin Legal Publishing (Texas)
Municipal Technical Advisory Service Institute for Public Service at the University of Tennessee (Tennessee)
Quality Code Publishing
ClerkBase
Ranson Citycode
State of Connecticut Judicial Branch Law Library Services
Drake University Law Library
Nebraska Access

Specific details on construction of each measure

The standard approach for each measure construction (denoted as "Standard construction" in the "Notes" column of Table S2) is as follows: the measure is created by finding a positive initial match in the text via a regular expressions search. After a positive match is found, the preceding and following 250 characters of the matching word or phrase are captured, creating a text string of roughly 500 characters. Next, for each captured text string corresponding to each measure, we search this text string for words or phrases with associated values in the "Weighted match" column. If the sum of the matching words in this text string exceeds 5, we set the indicator for the measure to 1. Otherwise, the indicator for the measure remains 0.

The collection of dimensional requirements (minimum lot size, maximum permitted density, building heights, and parking requirements) required a different procedure (denoted as "Dimensional requirements approach" in the "Notes" column of Table S2). We extracted this information directly from the text and dimensional tables. The process for extracting dimensional requirements from the text has the following steps:

- Begin with a match on a word or phrase in the "Initial match" column, capturing a range of preceding and following characters (minimum of 250, up to 2,000)
- Within the initial match, if a stop-word or phrase is not triggered, search for the relevant dimensional information as specified in the "Weighted match" column (acres, square feet, units per acre, parking spots per unit, or building heights in feet or stories) and extract the preceding and following 250 characters (70 for parking and building heights) for additional false-positive matches depending on the matched metric.
    - If acres, screen out strings relevant to planned unit developments or related developments (e.g., cluster developments), square feet and maximum density, or correctly parse mentions of units per acre.
    - If square feet, screen out strings relevant to planned unit developments or related developments (e.g., cluster developments)
- Next, limit the matched string to a narrower range of characters
    - If acres, the preceding and following 60 characters
    - If square feet, the preceding and following 65 characters
    - If units per acre, at least the preceding and following 25 characters, up to 65 characters
    - This step is skipped for parking requirements and building heights since their initial matched string is already smaller
- Within this narrower text string, conduct a series of tests for stop-words and stop-phrases along with indicators of a true-positive match
    - If acres, screen out matches relating to maximum lot size or maximum density and search for indications of minimum lot size, minimum lot area, etc.
    - If square feet, screen out matches relating to minimum floor size or unit size and search for indications of minimum lot size, minimum lot area, etc.
    - If units per acre, parking requirements, or building heights, screen out stop-words and stop-phrases

- If no stop-words or stop-phrases have been triggered and a match indicating minimum lot size or maximum permitted density was made in the second, narrower text string, create one last text string immediately surrounding the matched dimensional criteria
    - If acres, restrict the final string to the preceding and following 10 characters, screening out matches on maximum lot size information
    - If square feet, restrict the final string to at least the 10 (up to 45) preceding and following characters
    - If units per acre, restrict the final string to at least the 25 (up to 65) preceding and following characters
    - If parking, restrict the final string to the preceding and following 45 characters
    - If building heights, the preceding and following 10 characters
- Within this final matched string, extract any numbers, screening out irrelevant numeric information (e.g., lot width in feet). These numbers are stored as final matches and will be used to create the final indicators.

The process for extracting dimensional requirements from tables has the following steps:

- There are two different kinds of tables that can appear in municipal codes. In one format, the columns represent dimensional requirements and each row generally represents a zoning district (what we will call format A). In the other format, the columns represent zoning districts and the rows represent the dimensional requirements (what we will call format B). The general approach to parsing information is similar for both formats, with some exceptions which we note below. Also note that we do not explicitly extract parking information from dimensional regulation tables.
- Begin with a match on a word or phrase in the "Initial match" column, capturing a range of preceding and following characters (minimum of 250, up to 2,000). Generally, these strings will collect up to 2,000 characters following the match in order to capture potentially large dimensional regulations tables.
- Within this matched string, search for potential columns, representing either dimensional requirements in format A or zoning districts in format B and check for stop-words or stop-phrases.
- If enough potential columns exist, build the table header. Start with identifying where the rows begin by searching for either zoning districts in format A or dimensional requirements in format B. This will indicate the end of the table header. Calculate the beginning of the table header by iterating through matching words commonly indicating the beginning of a table (e.g., zoning district).
- If a valid header is constructed and no stop-words or stop-phrases are triggered, clean the header and construct the rows, verifying that each row is not too short or long. Loop through the rows to search for relevant dimensional requirements. Convert the row to a blank string if it includes irrelevant information or end the entire process for the original matched string if a particular stop-word or stop-phrase is triggered (e.g., notes:)
- If the table is in format A, identify columns with minimum lot size, maximum permitted density and/or maximum building height information via regular expressions searches. Create a list version of the table header, marking the index of the column with the relevant dimensional criteria. The number stored in the matching index for eligible rows (i.e., single units in residential zoning districts) will be extracted as a final match.

- If the table is in format B, identify the rows that contain minimum lot size, maximum permitted density and/or maximum building height information. Create a list version of the header, noting the first index indicating inappropriate matches (e.g., PUDs, cluster developments, commercial, industrial). Do not allow matches for this and future indices, unless the information pertains to a single residential unit. The eligible numeric information of the flagged row will be stored as final matches.

Finally, the process for determining the proportion of residential districts that permit multi-family housing by right is as follows:

- Begin with a match on a word or phrase in the "Initial match" column, capturing a range of preceding and following characters up to 2,000.
- Search this text string for any possible residential district, exiting the process if a stop-word or stop-phrase is triggered.
- If a match is found, search the text string for indications of single-family, multi-family, two or three-family (including townhomes and attached single-family), or mixed-use permissions. Calculate distances from the matched residential district indicator to matching instances of these permitted uses and assign the matching district to the permitted use with the smallest distance from the matched indicator in the string. If a flag is triggered indicating that multiple permitted uses are present in the text (indicating that distance to the original match is not a valid approach), assign the permitted use via the following hierarchy: any multi-family match indicates permitted multi-family use, followed by mixed-use, followed by two-family, followed by single-family. For instance, if a district R-3 indicates that single-family, two-family and multi-family housing is permitted by right, the district will be assigned as a multi-family permitting district for this particular string.
- Store each matching residential district as key in a dictionary along with each one of their matching permitted uses. After completing this process for all input strings, assign the matched residential districts to their modal values. So, for instance, suppose districts R-1, R-2, and R-3 were matched with the following values: [R-1: single-family, single-family, two-family; R-2: two-family, two-family; R-3: multi-family, single-family, multi-family]. R-1 will be assigned single-family, R-2 two-family, and R-3 multi-family. In the case of ties, the first modal value calculated is assigned.
- Finally, calculate the proportion of residential districts permitting multi-family housing by right. The numerator will be the number of matched districts assigned to either multi-family or mixed-use. The denominator will be all matched residential districts with at least one value. In the example above, the final value would be 1/3.

See Table S2 below for information regarding matching words and weights, along with additional notes on measure construction.

Table S2. Matching words and weights for measure construction

Measure	Initial match	Weighted match (weight value in parentheses)	Notes
Restrict single-family permits	residential subdivision building permits, unit ceiling, growth management, growth control, growth rate, development approvals	Limit (1), growth management (3), growth control (3), growth (1.5), scheduled development (1), maximum (2), no more (1), population (1), annual (1), year (1), fixed (2), controlled (2), quota (2), moratorium (2), allocate (2), cap (2), approved (1), calculation (1)	Standard construction; we manually correct false positive flags for four municipalities (Orlando, FL; Palm Beach Gardens, FL; Winstom-Salem, NC; Barre, MA)
Restrict multi-family permits	residential subdivision building permits, unit ceiling, growth management, growth control, growth rate, development approvals	Limit (1), growth management (3), growth control (3), growth (1.5), scheduled development (1), maximum (2), no more (1), population (1), annual (1), year (1), fixed (2), controlled (2), quota (2), moratorium (2), allocate (2), cap (2), approved (1), calculation (1)	Standard construction; we manually correct false positive flags for four municipalities (Orlando, FL; Palm Beach Gardens, FL; Winstom-Salem, NC; Barre, MA)
Limit single-family units	unit ceiling, growth management, growth control, growth rate, development approvals	Construction (1), unit (1), dwelling (1), limit (1), growth management (3), growth control (3), growth (1.5), scheduled development (2), maximum (2), population (1), annual (1), year (1), fixed (2), controlled (1), quota (1), moratorium (2), allocate (1), cap(2), approved (1)	Standard construction; we manually correct false positive flags for four municipalities (Orlando, FL; Palm Beach Gardens, FL; Winstom-Salem, NC; Barre, MA)
Limit multi-family units	unit ceiling, growth management, growth control, growth rate, development approvals	Construction (1), unit (1), dwelling (1), limit (1), growth management (3), growth control (3), growth (1.5), scheduled development (2), maximum (2), population (1), annual (1), year (1), fixed (2),	Standard construction; we manually correct false positive flags for four municipalities (Orlando, FL; Palm Beach Gardens, FL; Winstom-Salem, NC; Barre, MA)

		controlled (1), quota (1), moratorium (2), allocate (1), cap (2), approved (1)	
Limit multi-family dwellings	unit ceiling, growth management, growth control, growth rate, development approvals	Restricted (1), allowable (1), limit (1), growth management (3), growth control (3), growth (1), scheduled development (1), maximum (2), no more (1), population (1), fixed (1), controlled (2), quota (2), moratorium (2), allocate (1), annual (1), year (1), cap (2), approved (1), calculation (1), dwellings (1)	Standard construction; we manually correct false positive flags for three municipalities (Orlando, FL; Winstom-Salem, NC; Barre, MA)
Limit multi-family dwelling units	unit ceiling, dwelling units per building, growth management, growth control, growth rate, development approvals, minimum additional lot area	Restricted (1), allowable (1), limit (1), growth management (3), growth control (3), growth (1), scheduled development (1), maximum (2), no more (1), population (1), fixed (1), controlled (2), quota (2), moratorium (2), allocate (1), annual (1), year (1), cap (2), approved (1), calculation (1), dwelling units (1)	Standard construction; we manually correct false positive flags for two municipalities (Orlando, FL; Winstom-Salem, NC)
Minimum lot size	lot area, lot size, lot, area of parcel, zoning district, residential zones, residential district, residential district r-1 residential district r-a, schedule of, residential r-2 district, residential r-3, district, residential r-4 district, dimension regulations, lot require, lot yard and density regulations, dimensional regulations, dimensional require, dimensional and density regulations, development standards, intensity of use, dimensional control, zone dwelling family size, intensity regulations, dimensional standards, dimension restrictions, parcel size, maximum density, density, minimum building site area, bulk and replacement',	Minimum (3), lot size (2), lot area (2), lot (1), building area (2), acreage (2), shall be (1), require (1)<br><br>For particular minimum lot sizes, match on the following terms:<br>Acre, ac., ac, square feet, sf, s.f., sq.feet, sq ft, square, for each dwelling unit,<br>sq. feet, per dwelling unit<br><br>Additional search terms for dimensional tables: minimum lot area per dwelling unit, area per dwelling unit,	For acres, matches above 5 are not allowed unless the context suggests the text refers to rural or agricultural districts, in which case matches of up to 50 acres are permitted.<br><br>For square feet, matches below 2,000 are not permitted, unless the context specifies a per-unit measure. Matches below 1,000 or above 217,800 are not permitted in any circumstance. If the context suggests the text refers to multi-

	maximum unit allowed, height and area regulations, area and bulk schedule, area and bulk standards, district design require, height and area require, lot and bulk standards, height and lot require, area setback and height require, height area and yard require, bulk and area regulations, density schedule, dimensional table, height and yard require, bulk and yard regulations, spatial require, zoning district schedules, lot standards by zone, development regulations, lot dimension and intensity standards,<br>density and bulk require, bulk regulations, bulk and placement regulations, minimum lot size per dwelling unit, bulk and coverage controls, bulk require, land space require, lot area frontage and yard require, yard and height require, lot standards matrix, area yard and height standards, area yard and height regulations, other dimensions and space require, area, yard and height regulations, bulk and area standards, development criteria district, zone standards, height limit lot sizes and coverage, land use district and allowable uses, summary of zoning district require, bulk and setback regulations, residential bulk chart, bulk matrix, bulk yard and space require, residential uses and require, zoning district regulation chart, density regulations, standards for principal buildings on individual lots, lot and yard require, lot yard area and height require, area yard and height require, 'site dimensions, density dimensions and other standards, districts, density and intensity limit, bulk schedules	minimum lot area per family, minimum lot area per dwelling, minimum lot area per du, area of lot, area/du, minimum lot area per unit, lot area per unit, minimum lot size, min. lot area, minimum lot area, minimum lot, lot minimums, lot area, minimum size lot per unit, lot area minimum, minimum size lot, minimum size per zoning lot, lot size, area in square feet per additional family, square feet, square feet per dwelling unit, square feet/dwelling unit, square feet per additional family, area in square feet,<br>area, per family, per unit, minimum lot require, maximum density, single family, minimum parcel size, minimum net site area, lot areas<br><br>Additional search terms for in-text:<br>1 du/, 1 dwelling unit per, 1 dwelling per, 1 unit per x acres, minimum, not less, no less, lot size, lot area, lot surface area,<br>lot with septic, r-x residential, area:, 1 unit/ x acres, 1 unit to x acres, single family dwelling,<br>lot shall have an area of not less than, an area of not less than, zoning lots not less than,<br>no zoning lot less than, 1 dwelling unit per existing lot, total area of each lot not less than, no lot shall be created which contains, in no	family housing, matches over 15,000 are not permitted.

		case will a lot be platted with less than, residential dwelling unit on x acres, residential dwelling on x acres, land area per dwelling, shall contain at least, residential dwelling unit on x acres, residential dwelling on x acres	
Maximum permitted density	lot area, lot size, lot, multiple dwelling, zoning district, residential district, residential district r-1, height and lot require, zoning district schedules, residential district r-a, residential r-2 district, residential r-3 district, residential r-4 district, land area provide for each dwelling unit, dimensional table, dimension regulations, dimensional regulations, dimensional require, zone dwelling family size, lot yard and density regulations, area setback and height require, spatial require, dimensional and density regulations, intensity of use, dimensional controls, area and bulk standards, development standards, intensity regulations, dimensional standards, dimension restrictions, schedule of, maximum permitted residential density, maximum allowable residential density, maximum permitted density, maximum allowable density, maximum density, density, residential acreage dwelling unit, lot require, height and area regulations, height and yard require, height area and yard require, bulk yard and space require,<br>multi family, density schedule, maximum unit allowed, residential uses and require, acre\/dwelling unit, per dwelling unit, for each dwelling unit, bulk and replacement, dwelling unit per acre, square feet\/dwelling unit, unit\/net acre, district design require, height and area require, lot and bulk standards, bulk require, bulk and yard regulations, density regulations,	For particular maximum permitted densities, match on the following terms:<br><br>Dwelling unit per net acre, unit per net acre, dwelling unit per acre, dwelling unit per each 1 net acre, dwelling unit per acre, unit per acre, unit/net acre, unit per gross acre, unit per net platted acre, du/gross acre, maximum dwelling unit per structure, maximum dwelling unit per gross acre, density per acre, maximum dwelling unit per buildable acre, up to x units, up to x dwelling units, square feet/du, x dwelling units, dwelling per gross acre, minimum number of unit, dwelling unit, dua<br><br>Additional search terms for dimensional tables: maximum density, maximum net density	For square feet, matches below 2,000 are not permitted, unless the context specifies a per-unit measure. Matches below 380 are not permitted in any circumstance. If the context suggests the text refers to multi-family housing, matches over 15,000 are not permitted.<br><br>For units per acre, matches above 165 are not permitted.<br><br>If no maximum permitted density information is collected, the information is imputed from minimum lot size information.

	bulk and area regulations, lot standards by zone, summary of zoning district require, development regulations, lot dimension and intensity standards, density and bulk require, bulk regulations, bulk and placement regulations, minimum lot size per dwelling unit, land space require, lot area frontage and yard require, yard and height require, lot standards matrix, area yard and height standards, area yard and height regulations, bulk and coverage controls, density dimensions and other standards, other dimensions and space require, area, yard and height regulations, bulk and area standards, development criteria district, zone standards, height limit lot sizes and coverage, land use district and allowable uses, zoning district regulation chart, site dimensions, bulk and setback regulations, residential bulk chart, bulk matrix, standards for principal buildings on individual lots, area and bulk schedule, lot and yard require, lot yard area and height require, area yard and height require, districts:, density and intensity limit, bulk schedules		
Open space requirements	Open space	open space (2), at least (1), no less (1), minimum amount (1), minimum of (1), percent (1), in lieu (1.5), set aside (1), pay (1), fee (1), preserve (1), require amount (1.5), require (1), contribute (1), dedicate (1), reserve (1), provide (1), devoted (1)	Standard construction
Inclusionary zoning programs	Inclusionary, affordable, mixed income housing, low cost housing	Inclusionary (3), affordable (3), affordability (2), mixed income housing (2.5) nonprofit housing (2.5) workforce housing (2.5), low cost housing (2.5), set aside (2),in lieu (2), defer (2), waive (2), waiver (2), impact fee (2), housing	Standard construction

		fee (2), density bonus (2.5), density bonuses (2.5), increase in density (2.5), density may be increased (2.5), additional densities (2.5), density increases (2.5), above the base density (2.5), height increase (2.5), require (1), reserve (1), refund (1), exempt (2),shall not apply (2), unit per acre (2), shall not be required (2), incentives (2), taxation (1), payments (1), parking (1), space per dwelling unit (0.5), additional (1), height (1.5), at least (1.5), deed restricted (1), percent (1)	
City council approval – no rezoning	city council, town council, village council, village board, township council, the council, board of aldermen, city commission, borough council, board of selectmen, board of supervisors, governing body, board of commissioners, board of mayor and aldermen, mayor and council, board of trustees	use permit (2), building permit (2), zoning permit (2), construction permit (2), improvement location permit (2), special permit (2), site plan review (2), site plan approval (2), special permit granting authority (2), conditional use approval (2), conditional permitted use (2), design review (2), public hearing (2), developer's agreement (2), development agreement (2), development agreements (2), type ii (2), type 2 (2), type iii (2), type 3 (2), conditional use (1), plat (1), planned unit development (1), review (1), report (1), application (1), construction (1), issue (1), recommend (1), ,recommendation (1), recommendations (1), approve (1), grant (1), certification (1), plan	Standard construction

		(1), fee schedule (-1), schedule of fees (-1)	
Planning commission approval – no rezoning	planning board, planning commission, planning and zoning commission, planning and zoning board, planning and appeals commission, plan commission, planning and sustainability commission, redevelopment board, zoning commission, land use board, the commission, metropolitan development commission, development commission	use permit (2), building permit (2), zoning permit (2), construction permit (2), improvement location permit (2), special permit (2), site plan review (2), site plan approval (2), special permit granting authority (2), conditional use approval (2), conditional permitted use (2), design review (2), public hearing (2), developer's agreement (2), development agreement (2), development agreements (2), type ii (2), type 2 (2), type iii (2), type 3 (2), conditional use (1), plat (1), planned unit development (1), review (1), report (1), application (1), construction (1), issue (1), recommend (1), ,recommendation (1), recommendations (1), approve (1), grant (1), certification (1), plan (1), fee schedule (-1), schedule of fees (-1)	Standard construction
County board approval – no rezoning	county board of commissioners, county board, county commissioners, county board of supervisors, county commission, county council, parish board of commissioners, parish board, parish commissioners, parish board of supervisors, parish commission, parish council, board of freeholders, board of chosen freeholders	use permit (2), building permit (2), zoning permit (2), construction permit (2), improvement location permit (2), special permit (2), site plan review (2), site plan approval (2), special permit granting authority (2), conditional use approval (2), conditional permitted use (2), design review (2), public hearing (2), developer's agreement (2), development	Standard construction

		agreement (2), development agreements (2), type ii (2), type 2 (2), type iii (2), type 3 (2), conditional use (1), plat (1), planned unit development (1), review (1), report (1), application (1), construction (1), issue (1), recommend (1), ,recommendation (1), recommendations (1), approve (1), grant (1), certification (1), plan (1), fee schedule (-1), schedule of fees (-1)	
Public health board approval – no rezoning	health department, department of health, public health board, public health commission	use permit (2), building permit (2), zoning permit (2), construction permit (2), improvement location permit (2), special permit (2), site plan review (2), site plan approval (2), special permit granting authority (2), conditional use approval (2), conditional permitted use (2), design review (2), public hearing (2), developer's agreement (2), development agreement (2), development agreements (2), type ii (2), type 2 (2), type iii (2), type 3 (2), conditional use (1), plat (1), planned unit development (1), review (1), report (1), application (1), construction (1), issue (1), recommend (1), ,recommendation (1), recommendations (1), approve (1), grant (1), certification (1), plan (1), fee schedule (-1), schedule of fees (-1)	Standard construction

Design review board approval – no rezoning	site plan and architectural review board, site plan and architectural review commission, site plan and architectural review committee, architectural review board, architectural review commission, architectural review committee, site plan review board, site plan review commission, site plan review committee, design review board, design review commission, design review committee, design board, design commission, design committee, development review board, development review commission, development review committee, visual resources review board	use permit (2), building permit (2), zoning permit (2), construction permit (2), improvement location permit (2), special permit (2), site plan review (2), site plan approval (2), special permit granting authority (2), conditional use approval (2), conditional permitted use (2), design review (2), public hearing (2), developer's agreement (2), development agreement (2), development agreements (2), type ii (2), type 2 (2), type iii (2), type 3 (2), conditional use (1), plat (1), planned unit development (1), review (1), report (1), application (1), construction (1), issue (1), recommend (1), ,recommendation (1), recommendations (1), approve (1), grant (1), certification (1), plan (1), fee schedule (-1), schedule of fees (-1)	Standard construction
Environmental review board approval – no rezoning	environmental review board, environmental review committee, environmental commission, environmental impact review board, environmental impact review committee, environmental review advisory board, environmental review advisory committee, environmental assessment board, environmental assessment committee	use permit (2), building permit (2), zoning permit (2), construction permit (2), improvement location permit (2), special permit (2), site plan review (2), site plan approval (2), special permit granting authority (2), conditional use approval (2), conditional permitted use (2), design review (2), public hearing (2), developer's agreement (2), development agreement (2), development	Standard construction

		agreements (2), type ii (2), type 2 (2), type iii (2), type 3 (2), conditional use (1), plat (1), planned unit development (1), review (1), report (1), application (1), construction (1), issue (1), recommend (1), ,recommendation (1), recommendations (1), approve (1), grant (1), certification (1), plan (1), fee schedule (-1), schedule of fees (-1)	
City council approval – rezoning	city council, town council, village council, village board, township council, the council, board of aldermen, city commission, borough council, board of selectmen, board of supervisors, governing body, board of commissioners, board of mayor and aldermen, mayor and council, board of trustees	Amendment (2.5), amend (2.5), amended (2.5), variance (2.5), variation (2.5), special exception permit (2.5), rezoning (2.5), type iv* (2), type 4 (2), application (1), public hearing (2), approve (1), grant (1), provide (1), authorize (1), recommend (1), recommendations (1), final action (1), take action (1), fee schedule (-1), schedule of fees (-1)	Standard construction
Planning commission approval – rezoning	planning board, planning commission, planning and zoning commission, planning and zoning board, planning and appeals commission, plan commission, planning and sustainability commission, redevelopment board, zoning commission, land use board, the commission, metropolitan development commission, development commission	Amendment (2.5), amend (2.5), amended (2.5), variance (2.5), variation (2.5), special exception permit (2.5), rezoning (2.5), type iv* (2), type 4 (2), application (1), public hearing (2), approve (1), grant (1), provide (1), authorize (1), recommend (1), recommendations (1), final action (1), take action (1), fee schedule (-1), schedule of fees (-1)	Standard construction
Zoning board approval – rezoning	zoning board, board of zoning appeals, board of appeals, board of appeal, board of adjustment and appeals, board of adjustment, zoning hearing board, adjustment	Amendment (2.5), amend (2.5), amended (2.5), variance (2.5), variation (2.5), special exception permit (2.5), rezoning (2.5), type iv*	Standard construction

	board, adjustment commission, adjustment committee	(2), type 4 (2), application (1), public hearing (2), approve (1), grant (1), provide (1), authorize (1), recommend (1), recommendations (1), final action (1), take action (1), fee schedule (-1), schedule of fees (-1)	
County board approval – rezoning	county board of commissioners, county board, county commissioners, county board of supervisors, county commission, county council, parish board of commissioners, parish board, parish commissioners, parish board of supervisors, parish commission, parish council, board of freeholders, board of chosen freeholders	Amendment (2.5), amend (2.5), amended (2.5), variance (2.5), variation (2.5), special exception permit (2.5), rezoning (2.5), type iv* (2), type 4 (2), application (1), public hearing (2), approve (1), grant (1), provide (1), authorize (1), recommend (1), recommendations (1), final action (1), take action (1), fee schedule (-1), schedule of fees (-1)	Standard construction
County zoning authority	county zoning board', county zoning commission', county planning board, parish zoning board, parish zoning commission, parish planning board	Amendment (2.5), amend (2.5), amended (2.5), variance (2.5), variation (2.5), special exception permit (2.5), rezoning (2.5), type iv* (2), type 4 (2), application (1), public hearing (2), approve (1), grant (1), provide (1), authorize (1), recommend (1), recommendations (1), final action (1), take action (1), fee schedule (-1), schedule of fees (-1)	Standard construction
Town meeting approval – rezoning	Town meeting	Amendment (2.5), amend (2.5), amended (2.5), variance (2.5), variation (2.5), special exception permit (2.5), rezoning (2.5), type iv* (2), type 4 (2), application (1), public hearing (2), approve (1), grant (1), provide (1), authorize (1),	Standard construction

		recommend (1), recommendations (1), final action (1), take action (1), fee schedule (-1), schedule of fees (-1)	
Environmental review board approval – rezoning	environmental review board, environmental review committee, environmental commission, environmental impact review board, environmental impact review committee, environmental review advisory board, environmental review advisory committee, environmental assessment board, environmental assessment committee	Amendment (2.5), amend (2.5), amended (2.5), variance (2.5), variation (2.5), special exception permit (2.5), rezoning (2.5), type iv* (2), type 4 (2), application (1), public hearing (2), approve (1), grant (1), provide (1), authorize (1), recommend (1), recommendations (1), final action (1), take action (1), fee schedule (-1), schedule of fees (-1)	Standard construction
ADU	accessory dwelling unit, accessory dwelling units, accessory apartment, accessory apartments, accessory dwelling, accessory dwellings, accessory suite, accessory suites, ancillary unit, ancillary units, basement apartment, basement apartments, carriage house, carriage homes, carriage houses, carriage homes, garden cottage, garden cottages, granny cottage, granny cottages, granny unit, granny units, secondary suite, secondary suites, granny flat, granny flats, guest house, guest houses, backyard cottage, backyard cottages, in-law unit, in-law units, in-law suite, in-law suites, in-law flat, in-law flats, secondary unit, secondary units, secondary dwelling unit, secondary dwelling units, laneway house laneway houses, secondary dwelling unit, secondary dwelling units	Permitted (5.5), permit (5.5), p (5.5), shall be (2.5), containing (1.5), detached (1), attached (1), free-standing (1)	Standard construction
Maximum permitted height	maximum height, building height, height	For particular building heights, match on the following terms: feet, ft, stories, story<br><br>Additional search terms for dimensional tables:	Matches below 10 feet are not permitted. Matches above 165 feet are not permitted in the case of searching dimensional tables.

		Maximum height, building height, maximum bldg. height, height, max ht, principal, feet, stories	Matches for stories are limited to 50 (format A) and 100 (format B) for dimensional tables.
Minimum parking requirements	parking spots, parking spaces, parking, off-street spaces require, minimum parking require, minimum spaces require, vehicle, one space for	For particular parking requirements match on the following terms:<br><br>parking space, parking spaces, parking spot, parking spots, parking, guest space, per du, per dwelling unit, per unit, minimum parking require, for each dwelling unit, for every dwelling unit, for each apartment, minimum spaces require, for each family, spaces	Matches above 10 parking spaces per unit are not allowed.<br><br>If no parking information is found via the regular process, we conduct additional regular expression searches.
Permit multi-family by right	residential districts, residential district, residential single-family district, residential multi family district, residential single family district, single residential, multiple residential, density residential, zoning district schedules, three-family district, residential detached zones, residential multi family district residential multiple-family district, residential zone, residential multi family district, zoning districts, zoning district, zone district, multi family residential, single family residential, land use districts, residential high density, residential medium density, rural residential, residential one acre, residential two acre, housing district, residence zone, residential zone, dwelling zone, multiuse zone, mid-rise district, high-rise district, mixed use zone, overlay district, use regulation schedule, housing (four stories or less) district, residential overlay, one-family zone, multi family zone, classes of districts, district, district that is designed to, residence districts, residential	single family residence district, duplex residence district, sfrt, multiple residence district, res-x, r-x-x. xr-x, xr-x, ag, ae, rhd, re-x, rx-x, gr-x, sr-x, rax, rbx, ah-x, ga-x, th, rco, orc, rcd, rcm, ra, rex, rtx, rp-x, r-xmh, m-u-x, r-m-x, r-md-sz, md-x, r-hd-sz, rd-x, rp, gr, su, 1f-x, re, rd, rgx, rms, rmm, rcp, rc, ro, rnc, tld, tmd, thd, cd, rmf-x, rmf, rmh, r-x-e, rm-x, rs, r, srr, rr, rh, ru-x, rsh-x, src, srl, srh, rlx, rhx, r:x, sr, fr, rx-, sfr-x, sfr, mhr, vldr, tn-ldr, tn-mdr, mcn-ldr, mcn-mdr, mon-ldr, mon-mdr, ldr, sfa, url, ar-x, sf-x, 2f-x, mf-x, r/c, ot-mf, ot-sf, nrx, lrx, smu-slu, sm-x, ncx, mf-x, rm, rx, r-xx, os-x, otr, smf, emf, mmf, hr, mfx, mf, tf, mp, sn, rcr, ora, ira, slo, llrd, ldd, ld-r, md-r, mhd-r, hd-r, rs, lrr, lr-x, lr, ur-ld, ur-md, ur-hd, ur, ovr, vr-	The code includes a series of procedures to shorten or cut-off strings when necessary to avoid false-positive matches.<br><br>If no residential districts are found or no residential districts have any matching values, we conduct a series of additional regular expression searches.

	classifications, district regulations, creation of districts, dwelling district:, r1 district, r2 district, r3 district, r4 district, r5 district, r6 district, r7 district, r-1 district, r-2 district, r-3 district, r-4 district, residential-general district, low density district, medium density district, high density district, rural density district, general residence district, residential use district, residence district, residential urban district, residential suburban district, residential limited business district, re residential-existing district, conservation district, r-16 district, low density residential, medium density residential, rm district, low density-residential, medium-density residential, medium-high-density residential, r-1 residential., r-2 residential., r-3 residential., residence a district, residence aa district, residence b district, residence bb district, residence c-1 district, residence c-2 district, residence cc district, residence d district, residence dd district, residence e district, residence ee district, residence f district, residence ff district, residence k district, residence a-1, residence a-2, low-rise, medium-rise, high-rise,district, residential urban zone, residential flexible zone, urban residence, suburban residence	mf, vr, hrc, hr, mmh, scmxd, mxd, ru, rf, mxr, r-.x, h-x-x, nc, tnx, t-xx, tx, mhp, rmo, rso, rsf, rlm-x, rlm, r-ld, r-mf, r-mf, sfx, rmx, murzd, mu-r-x, mh-x, mh, mdr, hdr, dmu, mu, mn, tcmu, pdm, tr, lhc, lha, rb, oh, rth, arzd, rzd, vzd, dm-x, ul-x, um-x, uh-x, mc, mr, ns, nu, mx-x, dd-x, x-x, single family dwelling district, two family dwelling district, multi family dwelling district, a apartment, osx, a-a, exx, ex, ga, residential x, office/residential, crd, crd-x, residence a-x, residence c-x, residence e, residence f, residenca aaa, residence aa, residence a, o residential use district, residence x, class ux, residential x, ceod, un-x, ddh, mdrd, x single family district, low-rise apartment district, high-rise apartment district, nrd, rdd, ub, aho-x, rt, tr-x, cr-x, ce-x, cdd, mfrd, mfr-x, cr, neighborhood, urban, village residential, residential-commercial, general residence district, residence x, x-acre single family district, x square foot single family, low-rise multi family, medium rise multi family, family district, a residence districts, b residence districts, r residential district, district x, x residence districts, x residential districts, district x, harbor village district, traditional village district, multiple dwelling district, village	

		district, rural residential district, general residential district, rural district, medium density district, affordable housing overlay zone, affordable housing overlay district, multiple dwelling district, dwelling district, garden apartment district, residential-general, rural residential, x district, low-density residential, medium-density residential, high-density residential, residential district x, residential district, residence district, two family residence district, x dwelling district, multi family residence district	

Table S3. ZRI loadings on subindices and correlations

Subindex	Reduced Zoning Restrictiveness Index		Full Zoning Restrictiveness Index	
	Loading	Correlation with Zoning Restrictiveness Index	Loading	Correlation with Zoning Restrictiveness Index
Explicit Growth Controls Subindex (EGCI)	0.23	0.30	0.22	0.34
Open space requirements Subindex (OSRI)	0.52	0.52	0.42	0.50
Minimum Lot Size Subindex (MLSI)	0.39	0.65	0.31	0.61
No Rezoning Subindex (NZI)	0.40	0.47	0.28	0.38
Rezoning Subindex (RZI)	0.16	0.19	0.00	0.03
Maximum Permitted Density Subindex (MPDI)	-0.35	-0.52	-0.26	-0.44
Inclusionary Zoning Subindex (IZI)	0.48	0.47	0.50	0.58
Accessory Dwelling Unit Subindex (ADUI)	-	-	0.50	0.59
Maximum Height Subindex (MHI)	-	-	-0.01	0.0
Minimum Required Parking Subindex (MRPI)	-	-	-0.07	-0.11
Permitted Multifamily Housing Subindex (MFPI)	-	-	0.16	0.11

As the results in Table S3 detail, the Reduced ZRI loads positively and is positively correlated with all but one of the subindices, the MDPI. The OSRI and IZPI indices contribute the most to the Reduced ZRI, followed by the NZI index and the minimum lot size and density information captured by the MPDI and MLSI subindices. The EGCI and RZI subindices contribute the least to the Reduced ZRI, which in the case of EGCI, could be due to the lack of growth control implementation outside a handful of municipalities. As for RZI, it is conceivable that municipalities generally require a similar number of reviewing and approving authorities in the case of rezonings, but vary more in the extent to which they require reviews and approvals for projects that meet current zoning regulations.

Though a positive ZRI loading on IZPI and a negative loading on MPDI may initially seem counterintuitive, these results make sense in the context of previous research. Zoning

restrictiveness is understood to be more present in denser, oftentimes coastal housing markets. Inclusionary zoning programs heavily concentrate in these markets as well (see Figure S3), suggesting that these interventions could be born out of conditions that correlate with housing scarcity just as they may contribute to zoning restrictiveness.

Turning to the Full ZRI, the results for the loadings on the original subindices are generally similar. The contribution from the RZI is now negligible and contributions from other subindices such as the OSRI are reduced. Interestingly, the contribution from the IZI subindex seems more pronounced and on par with the ADUI. The contributions from maximum permitted building heights (MHI) and minimum parking requirements (MRPI) seem negligible and the subindex measuring the proportion of residential districts allowing multi-family housing by right contributes modestly to the Full ZRI.

Five subindices (OSRI, MLSI, MPDI, IZI, and ADUI) are highly correlated with the overall ZRI. In order to explore these relationships in more detail, we take the mean of either one of these subindices or closely related measures for all MSAs and rank the top ten highest values for each of the five measures. We do this to illustrate how conclusions regarding MSA-level zoning and land use restrictiveness vary depending on each individual measure. Table S4 below contains these results.

Table S4. MSA rankings by means of subindices (means in parentheses)

Rank	Proportion of municipalities requiring open space	Proportion of municipalities with one or two acre maximum minimum lot sizes	Proportion of municipalities permitting only 0-4 dwelling units per acre	Proportion of municipalities with inclusionary zoning programs	Proportion of municipalities permitting ADUs
1	Provo-Orem, UT (1.0)	Bridgeport-Stamford-Norwalk, CT (0.91)	Bridgeport-Stamford-Norwalk, CT (0.27)	San Diego-Carlsbad-San Marcos, CA (1.0)	Provo-Orem, UT (1.0)
2	Springfield, MA (1.0)	Worcester, MA (0.91)	Manchester-Nashua, NH (0.18)	San Francisco-Oakland-Fremont, CA (0.96)	Seattle-Tacoma-Bellevue, WA (0.96)
3	Washington-Arlington-Alexandria, DC-VA-MD-WV (1.0)	Phoenix-Mesa-Glendale, AZ (0.83)	San Antonio-New Braunfels, TX (0.15)	Seattle-Tacoma-Bellevue, WA (0.93)	San Francisco-Oakland-Fremont, CA (0.96)
4	Seattle-Tacoma-Bellevue, WA (0.96)	Portland-South Portland-Biddeford, ME (0.82)	Hartford-West Hartford-East Hartford, CT (0.13)	Los Angeles-Long Beach-Santa Ana, CA (0.90)	Portland-Vancouver-Hillsboro, OR-WA (0.95)
5	Portland-South Portland-Biddeford, ME (0.94)	Hartford-West Hartford-East Hartford, CT (0.77)	Springfield, MA (0.10)	Riverside-San Bernardino-Ontario, CA (0.85)	Washington-Arlington-Alexandria, DC-VA-MD-WV (0.93)
6	Atlanta-Sandy Springs-Marietta, GA (0.93)	Riverside-San Bernardino-Ontario, CA (0.75)	Cincinnati-Middletown, OH-KY-IN (0.10)	Boston-Cambridge-Quincy, MA-NH (0.82)	Manchester-Nashua, NH (0.91)
7	Allentown-Bethlehem-Easton, PA-NJ (0.93)	Manchester-Nashua, NH (0.73)	Providence-New Bedford-Fall River, RI-MA (0.09)	New York-Northern New Jersey-Long Island, NY-NJ-PA (0.80)	Los Angeles-Long Beach-Santa Ana, CA (0.88)
8	Harrisburg-Carlisle, PA (0.93)	Providence-New Bedford-Fall River, RI-MA (0.71)	Des Moines-West Des Moines, IA (0.09)	Bridgeport-Stamford-Norwalk, CT (0.73)	Riverside-San Bernardino-Ontario, CA (0.85)
9	Dayton, OH (0.92)	Atlanta-Sandy Springs-Marietta, GA (0.67)	Worcester, MA (0.08)	Manchester-Nashua, NH (0.73)	Hartford-West Hartford-East Hartford, CT (0.85)
10	Columbus, OH (0.91)	Provo-Orem, UT (0.60)	Grand Rapids-Wyoming, MI	Portland-South Portland-Biddeford, ME (0.69)	Worcester, MA (0.82)

The results in Table S4 illustrate some commonalities across the measures. For instance, MSAs that register as highly restrictive on the ZRI (e.g., Seattle-Tacoma-Bellevue, WA MSA) appear

to have some of the highest values across several of these measures. However, other MSAs with far lower ZRI scores (e.g., Columbus, OH MSA, San Antonio-New Braunfels, TX MSA) appear throughout these listings. Therefore, while a particular subindex may correlate highly with the ZRI, relying on one subindex alone to capture something as complex as exclusionary zoning can lead to fairly different conclusions depending on the subindex. This suggests to us the necessity of combining these subindices into a summary index such as the ZRI.

Table S5. Logistic regression results for weighting

	Dependent variable:			
	In final sample			
	All municipalities (2005-2009)	Municipalities in an MSA (2005-2009)	All municipalities (2015-2019)	Municipalities in an MSA (2015-2019)
Total population	0.870***	0.615***	0.706***	0.511***
	(0.065)	(0.058)	(0.059)	(0.054)
Share of homes that are owner occupied	-4.033***	-3.358***	-4.418***	-3.355***
	(0.150)	(0.187)	(0.161)	(0.195)
Share of population 65 years of age and older	0.046	0.856*	-0.797**	0.420
	(0.336)	(0.456)	(0.372)	(0.477)
Share of population under 18 years of age	1.865***	2.397***	1.289***	1.972***
	(0.348)	(0.486)	(0.397)	(0.544)
White share of population	-0.764***	-0.711***	-0.835***	-0.969***
	(0.105)	(0.132)	(0.113)	(0.135)
Ln(median property value)	0.660***	0.410***	0.243***	-0.133*
	(0.041)	(0.053)	(0.053)	(0.069)
Share of population with 4-year college degree or more	2.811***	2.668***	3.769***	3.931***
	(0.170)	(0.203)	(0.192)	(0.229)
Midwest region			-0.363***	-0.195***
			(0.063)	(0.070)
South region			-0.661***	-0.580***
			(0.068)	(0.074)
West region			0.163**	0.498***
			(0.079)	(0.093)
Constant	-8.104***	-5.460***	-2.713***	1.039
	(0.490)	(0.642)	(0.617)	(0.798)
Observations	43,892	18,505	41,738	18,189
Log Likelihood	-8,397.696	-5,684.601	-7,906.555	-5,454.442
Akaike Inf. Crit.	16,811.390	11,385.200	15,835.110	10,930.880
Notes:				*p<0.1; **p<0.05; ***p<0.01

a. We implement a similar methodology to Gyourko, Saiz and Summers (2008) and Gyourko, Hartley and Krimmel (2021), using the same set of covariates to estimate the probability of selection into different samples for the time periods overlapping with 2005-2009. However, we also include Census region (Northeast as reference category) as an additional predictor of sample inclusion in the periods overlapping with 2015-2019.
b. Standard errors are in parentheses.
c. We divide total population by 100,000 for readability as in Gyourko, Saiz and Summers (2008).
d. Data come from 2005-2009 ACS 5-year estimates (retrieved from Social Explorer and using R package tidyensus) and 2015-2019 ACS 5-year estimates (retrieved using R packages tidycensus and tigris).
e. We drop all census county divisions (CCDs), precincts, and census-designated places (CDPs), with the exception of CDPs in Hawaii

Table S6. Summary statistics for ZRI (2019-2021) with and without survey weights

Sample	Min	25th pct.	Median	Mean	75th pct.	Max	St. Dev.	Observations
Full sample	-3.17	-0.70	-0.05	0.0	0.69	3.51	1.00	2,639
Full sample with national weights	-27.09	-1.07	-0.52	-0.46	0.19	10.42	0.97	2,639
MSA sample	-3.17	-0.54	0.13	0.12	0.77	3.51	0.99	2,080
MSA sample with MSA weights	-24.24	-0.96	-0.15	-0.21	0.52	12.15	1.00	2,080
Non-MSA sample	-3.17	-1.07	-0.51	-0.43	0.18	2.51	0.92	578
Absolute difference between unweighted and weighted MSA-level ZRI scores among MSAs with at least 10 municipalities	0.00	0.06	0.19	0.30	0.36	2.2	0.38	48

Notes: We take the additional step of stabilizing our weights to guard against extreme weights and to reduce the variance.

The results in Table S6 indicate that applying survey weights does not lead to dramatically different distributions of ZRI scores. The results reveal that less regulated municipalities are less likely to be included in the full and MSA samples. This is reflected in the down-weighting of ZRI scores in the weighted results. Moreover, the magnitude of the minimum and maximum ZRI scores is amplified in the weighted results. Finally, the differences between weighted and unweighted MSA-level ZRI scores among MSAs with at least 10 municipalities are generally minor.

Table S7. Comparison of individual components of the No Rezoning (NZI) and Rezoning (RZI) subindices with Wharton Residential Land Use Regulatory Index 2018 sample

Measure	All municipalities		Municipalities in both Wharton Residential Land Use Regulatory Index samples		
	National Zoning and Land Use Database (N=2,639)	Wharton Residential Land Use Regulatory Index 2018 (N=2,844)	National Zoning and Land Use Database values (N=874)	Wharton Residential Land Use Regulatory Index 2018 values (N=874)	Matching values (N= 874)
No rezoning – Council approval	0.81	0.56	0.85	0.55	0.56
No rezoning – Planning board approval	0.93	0.66	0.93	0.67	0.64
No rezoning – Site design review board approval	0.20	0.15	0.21	0.16	0.76
No rezoning – County board approval	0.14	0.07	0.01	0.04	0.96
No rezoning – Environmental review board approval	0.01	0.10	0.00	0.06	0.94
No rezoning – Public health board approval	0.05	0.11	0.05	0.06	0.90
Rezoning – Council approval	0.91	0.77	0.94	0.87	0.86
Rezoning – Planning board approval	0.97	0.81	0.96	0.87	0.85
Rezoning – Zoning board approval	0.72	0.57	0.73	0.52	0.59
Rezoning – County board approval	0.03	0.08	0.03	0.04	0.93
Rezoning – County zoning authority approval	0.02	0.10	0.01	0.04	0.95
Rezoning – Environmental review board approval	0.00	0.09	0.00	0.06	0.94
Rezoning – Town meeting approval	0.04	0.40	0.04	0.36	0.65

Table S8. Sociodemographic characteristics of municipalities and MSAs by Zoning Restrictiveness Index category

Measure	Municipalities by Zoning Restrictiveness Index category			MSAs by Zoning Restrictiveness Index category		
	Low (1st quartile)	Medium (Interquartile range)	High (4th quartile)	Low (1st quartile)	Medium (Interquartile range)	High (4th quartile)
Total population	19,267	42,200	79.816	496,393	1,031,923	2,684,687
Population density	2,087	2,420	2,746	218	307	811
Land area (square miles)	17.42	17.83	36.95	3,214.10	3,214.03	3327.49
Ethnoracial diversity (entropy)	0.64	0.74	0.83	0.84	0.87	1.00
Ethnoracial segregation (D)	-	-	-	0.13	0.15	0.21
Socioeconomic segregation (rank-order H)	-	-	-	0.07	0.08	0.09
Median household income	$59,799	$68,685	$78,431	$56,019	$60,273	$69,782
Median property value	$188,573	$251,521	$335,320	$173,276	$203,970	$303,795
Share of population with four-year college degree (or more)	0.26	0.33	0.39	0.28	0.30	0.34
Household poverty rate	0.15	0.12	0.11	0.14	0.13	0.12

Notes:

a. The entropy index measures diversity (higher scores indicate more diversity) and is defined as $E = \sum_{k=1}^{K} \frac{k_j}{t_j} \cdot \ln(\frac{t_j}{k_j})$ where k indexes the K ethnoracial groups, k_j is the population of group k in tract j and t_j is the total population of tract j (Holloway, Wright and Ellis, 2012). We use six ethnoracial groups in our calculations (AIAN, Asian, Black, Latinx, white and Other).

b. The divergence index (D) measures ethnoracial segregation (higher values indicate more segregation) and is defined as $D_i = \sum_{m=1}^{M} \pi_{im} log \frac{\pi_{im}}{\pi_m}$ where π_{im} is group m's proportion of the population in location i and π_m is group m's proportion of the overall population (Roberto, 2016). We use the same six ethnoracial groups as with our entropy scores.

c. Rank-order H refers to the rank-order information theory index, which measures income segregation (see Reardon et al., 2018).

Table S9. Summary statistics of additional zoning and land use measures

	Average maximum permitted height across districts with residential units	Permit accessory dwelling units (ADUs) in municipality	Average minimum number of parking units required per dwelling unit across districts with residential units	Proportion of residential districts permitting multi-family housing by right
Mean (standard deviation)	34 feet (11.57)	0.46 (0.50)	1.8 spaces per unit (0.94)	0.39 (0.23)
Observations	2,505	2,639	2,616	2,611

Table S10. Differences in Zoning Restrictiveness Index values across different index and sample constructions

MSA	Median Zoning Restrictiveness Index	Median Zoning Restrictiveness Index with additional measures	Zoning Restrictiveness Index suburban-central city gap	Zoning Restrictiveness Index combined (median and gap measures)	Zoning Restrictiveness Index combined (standardized)
San Francisco MSA: original sample	2.84	2.55	0.70	3.55	0.91
San Francisco MSA: full sample	2.97	2.86	1.06	4.02	1.21
Houston MSA: original sample	1.23	0.83	1.94	3.17	0.65
Houston MSA: full sample	1.04	0.69	2.46	3.50	0.85

Table S11. Comparison of most restrictive municipalities between full National Zoning and Land Use Database and Wharton Residential Land Use Regulatory Index 2018 samples

Rank	All municipalities			
	National Zoning and Land Use Database values (N=2,639)		Wharton Residential Land Use Regulatory Index 2018 values (N=2,844)	
	Municipality	ZRI	Municipality	WRLURI
1	West Windsor, NJ	3.51	Kingstree, SC	4.86
2	Redmond, WA	3.51	Hidalgo, TX	3.94
3	Santa Paula, CA	3.34	Menands, NY	3.69
4	Olympia, WA	3.34	Niagara, NY	3.50
5	Lawrence Township, NJ	3.09	Rush, PA	3.46
6	Barnstable, MA	3.09	Coral Springs, FL	3.35
7	Chelan, WA	3.09	Ridgewood, NJ	3.20
8	Kingsburg, CA	2.98	Mineola, TX	3.20
9	Tualatin, OR	2.96	Calabasas, CA	3.07
10	Rohnert Park, CA	2.93	Annapolis, MD	3.03

Note: An earlier version of this table incorrectly labeled the final column values as ZRI. The final column values reflect WRLURI values. The updated table has been corrected.

Table S12. Comparison of most restrictive municipalities among municipalities in National Zoning and Land Use Database, Wharton Residential Land Use Regulatory Index 2018 and National Longitudinal Land Use Survey 2019 samples

Rank	Municipalities across all samples			
	National Zoning and Land Use Database values (N=242)		Wharton Residential Land Use Regulatory Index 2018/ National Longitudinal Land Use Survey 2019 values (N=242)	
	Municipality	ZRI	Municipality	ZRI (parallel)
1	West Windsor, NJ	3.19	Burien, WA	2.01
2	Rohnert Park, CA	2.69	Decatur, GA	1.80
3	Portsmouth, RI	2.36	Andover, MA	1.80
4	Brentwood, CA	2.21	Everett, WA	1.80
5	Winston-Salem, NC	2.02	Mercer Island, WA	1.80
6	Issaquah, WA	1.88	University Place, WA	1.80
7	Palm Desert, CA	1.71	Euless, TX	1.79
8	Riverside, CA	1.71	Portland, OR	1.67
9	San Ramon, CA	1.71	Arcadia, CA	1.65
10	Kansas City, MO	1.71	Carson, CA	1.60

Notes:

1) The ZRI scores for these samples are slightly different since the index (and its parallel index with Wharton Residential Land Use Regulatory Index 2018 and National Longitudinal Land Use Survey 2019 values) was constructed amongst samples that are subsets of the full NZLUD and Wharton Residential Land Use Regulatory Index /National Longitudinal Land Use Survey samples.
2) An earlier version of this table reflected an error in calculating the parallel ZRI values for the comparison sample (final two columns). The updated table has been corrected.

Table S13. Comparison of most restrictive MSAs between full National Zoning and Land Use Database and Wharton Residential Land Use Regulatory Index 2018 samples

Rank	All MSAs					
National Zoning and Land Use Database (N=328)				Wharton Residential Land Use Regulatory Index 2018 (N=320)		
	MSA	Municipalities in data	ZRI	MSA	Municipalities in data	WRLURI
1	Trenton-Ewing, NJ	6	3.18	El Centro, CA	1	4.10
2	Oxnard-Thousand Oaks-Ventura, CA	7	2.59	Bloomington, IN	1	3.60
3	Bremerton-Silverdale, WA	3	2.17	Flagstaff, AZ	1	3.43
4	Atlantic City-Hammonton, NJ	5	2.13	Trenton-Ewing, NJ	2	2.90
5	York, PA	6	2.11	San Luis Obispo-Paso Robles, CA	2	2.83
6	Washington-Arlington-Alexandria, DC-VA-MD-WV	15	2,05	Santa Rosa-Petaluma, CA	5	2.48
7	New York-Northern New Jersey-Long Island, NY-NJ-PA	108	2.01	Santa Barbara-Santa Maria-Goleta, CA	2	2.23
8	Providence-New Bedford-Fall River, RI-MA	21	1.81	Vallejo-Fairfield, CA	2	2.16
9	Las Vegas-Paradise, NV	3	1.63	Amarillo, TX	1	2.11
10	Seattle-Tacoma-Bellevue, WA	274	1.60	Knoxville, TN	1	2.10

Table S14. Comparison of least restrictive municipalities between full National Zoning and Land Use Database and Wharton Residential Land Use Regulatory Index 2018 samples

Rank	All municipalities			
	National Zoning and Land Use Database (N=2,639)		Wharton Residential Land Use Regulatory Index 2018 (N=2,844)	
	Municipality	ZRI	Municipality	WRLURI
1	Burlington, CO	-3.17	Polk, IN	-2.64
2	Elsa, TX	-3.17	Perry, OH	-2.57
3	Hornell, NY	-2.92	Mexico, ME	-2.55
4	Clifton, TN	-2.92	West Chester, PA	-2.48
5	Vidor, TX	-2.92	Wakeman, OH	-2.45
6	Elkins, WV	-2.92	Alton, IN	-2.36
7	Corydon, IN	-2.76	Endicott, NY	-2.30
8	West Seneca, NY	-2.75	Rindge, NH	-2.29
9	Floresville, TX	-2.75	Massena, NY	-2.29
10	Richmond, IN	-2.72	Ferndale, MI	-2.28

Table S15. Comparison of least restrictive municipalities among municipalities in National Zoning and Land Use Database, Wharton Residential Land Use Regulatory Index 2018 and National Longitudinal Land Use Survey 2019 samples

Rank	Municipalities across all samples			
	National Zoning and Land Use Database (N=242)		Wharton Residential Land Use Regulatory Index 2018/National Longitudinal Land Use Survey 2019 values (N=242)	
	Municipality	ZRI	Municipality	ZRI (parallel)
1	South St. Paul, MN	-2.13	North Strabane, PA	-2.56
2	Springfield, TN	-2.11	Portsmouth, RI	-2.50
3	Monroeville, PA	-2.08	Hampton, PA	-2.16
4	Justice, IL	-1.74	Marshfield, MA	-2.05
5	Forest Hill, TX	-1.74	Forest Hill, TX	-1.88
6	Pinecrest, FL	-1.71	Upper Arlington, OH	-1.80
7	Forest Park, OH	-1.71	Topsfield, MA	-1.73
8	Round Lake, IL	-1.65	Warrington Township, PA	-1.73
9	Sachse, TX	-1.61	Sterling Heights, MI	-1.72
10	Lighthouse Point, FL	-1.53	Utica, MI	-1.70

Notes:

1) The ZRI scores for these samples are slightly different since the index (and its parallel index with Wharton Residential Land Use Regulatory Index 2018 and National Longitudinal Land Use Survey 2019 values) was constructed amongst samples that are subsets of the full National Zoning and Land Use Database and Wharton Residential Land Use Regulatory Index/National Longitudinal Land Use Survey samples.
2) An earlier version of this table reflected an error in calculating the parallel ZRI values for the comparison sample (final two columns). The updated table has been corrected.

Table S16. Comparison of least restrictive MSAs between full National Zoning and Land Use Database and Wharton Residential Land Use Regulatory Index 2018 samples

Rank	All MSAs					
National Zoning and Land Use Database (N=328)				Wharton Residential Land Use Regulatory Index 2018 (N=320)		
	MSA	Municipalities in data	ZRI	MSA	Municipalities in data	WRLURI
1	Corpus Christi, TX	5	-2.76	Lake Havasu City-Kingman, AZ	2	-1.94
2	Dubuque, IA	2	-2.63	Lubbock, TX	1	-1.82
3	Parkersburg-Marietta-Vienna, WV-OH	2	-1.95	Texarkana, TX-Texarkana, AR	1	-1.74
4	El Paso, TX	2	-1.94	Lake Charles, LA	2	-1.72
5	Sebastian-Vero Beach, FL	3	-1.82	Dothan, AL	1	-1.61
6	Jacksonville, FL	3	-1.78	Albany, GA	2	-1.52
7	Charleston-North Charleston-Summerville, SC	4	-1.74	Fort Smith, AR-OK	2	-1.49
8	St. Joseph, MO-KS	2	-1.72	Auburn-Opelika, AL	2	-1.31
9	Hickory-Lenoir-Morganton, NC	4	-1.72	Ames, IA	2	-1.21
10	Lynchburg, VA	2	-1.59	San Angelo, TX	1	-1.20

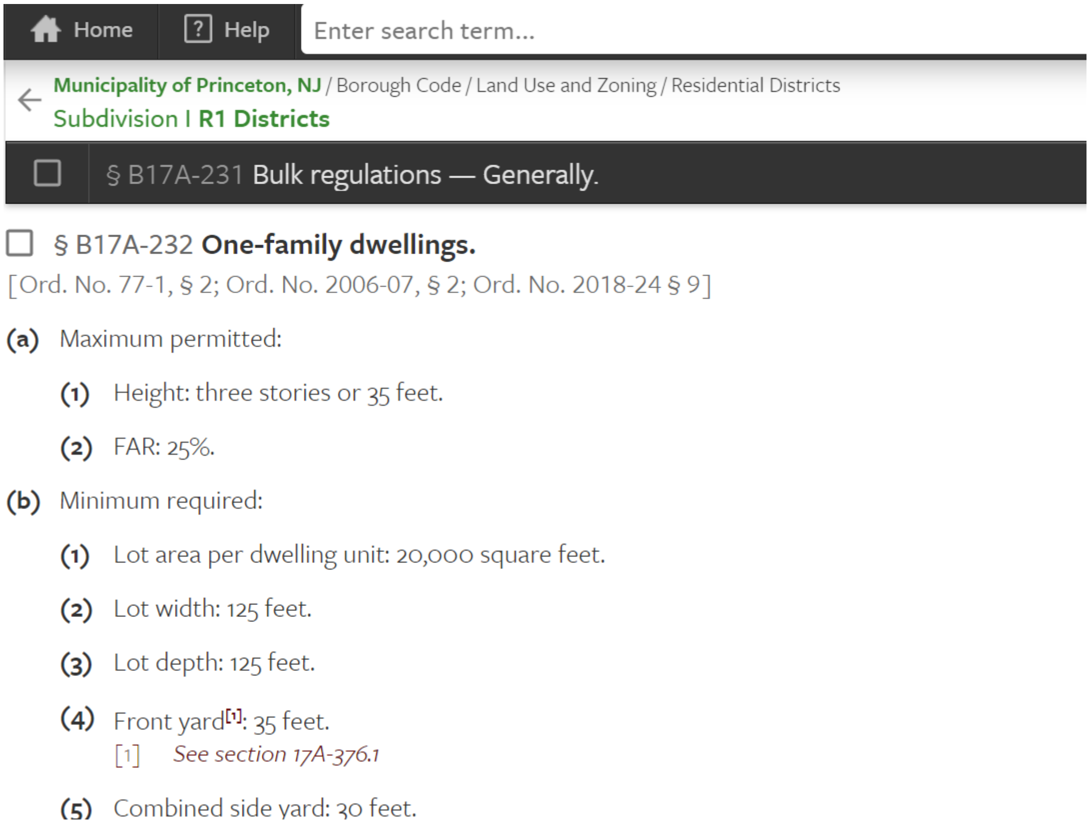

Home Help Enter search term...

Municipality of Princeton, NJ / Borough Code / Land Use and Zoning / Residential Districts
Subdivision I R1 Districts

§ B17A-231 Bulk regulations — Generally.

§ B17A-232 **One-family dwellings.**

[Ord. No. 77-1, § 2; Ord. No. 2006-07, § 2; Ord. No. 2018-24 § 9]

(a) Maximum permitted:

(1) Height: three stories or 35 feet.

(2) FAR: 25%.

(b) Minimum required:

(1) Lot area per dwelling unit: 20,000 square feet.

(2) Lot width: 125 feet.

(3) Lot depth: 125 feet.

(4) Front yard[1]: 35 feet.

[1] *See section 17A-376.1*

(5) Combined side yard: 30 feet.

Figure S1. Example of dimensional requirements stored in text

City of Trenton

Appendix A, Area and Bulk Schedules
Residential Districts

Zone	Use	Minimum Lot Size (square feet)	Minimum Yards[1] (feet)			Minimum Lot Width (feet)	Minimum Building Width (feet)	Maximum Building Height (stories/feet)
			Front[2]	Rear	Side			
RA	Detached 1-family dwelling units	6,000	Average or 30	35	One: 6<br>Both: 16	50	25	2.5/35
RB-1	Detached 1-family dwelling units	4,000	Average or 25	35	One: 6<br>Both: 14	40	20	2.5/35
RB-2	Detached 1-family dwelling units	4,000	Average or 20	35	One: 6<br>Both: 14	40	—	3/35
RB-2	Semidetached 1-family dwelling units	2,500 per unit	Average or 20	35	One: 6<br>Both: n/a	25 per unit	—	3/35
RB	Detached 1-family dwelling units	4,000	Average or 20	35	One: 6<br>Both: 14	40	—	3/35
RB	Semidetached 1-family dwelling units	2,500 per unit	Average or 20	35	One: 6<br>Both: n/a	25 per unit	—	3/35
RB	Row house dwelling units	1,500 per unit	Average or 20	35	—[3]	15 per unit	—	3/35

NOTES:

[1] See § 315-232, Yard exceptions.

[2] When there is only one existing structure on a block, the front yard setback for a new structure shall be as specified in the table.

[3] There shall be no more than eight units in any one row house structure. A minimum side yard setback of six feet is required for each end unit in a row house structure.

Figure S2. Example of dimensional requirements stored in tables

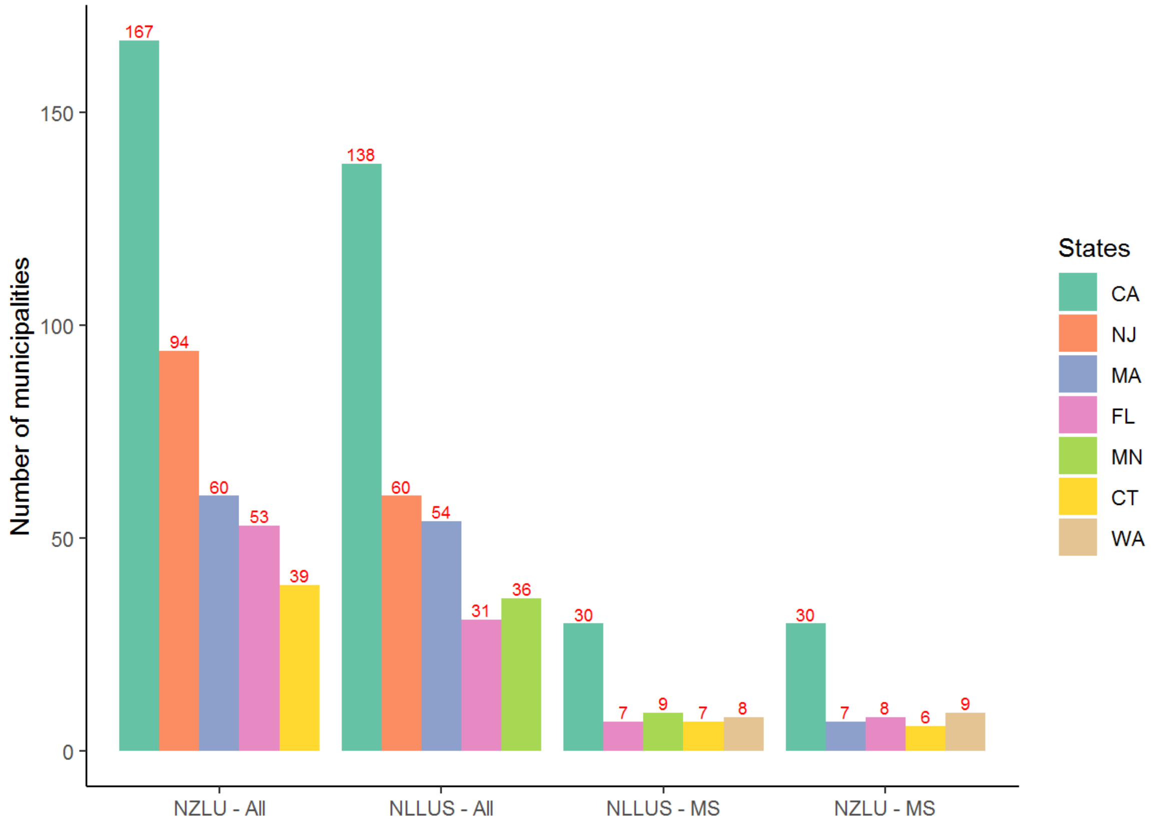

Note: MS: matching subset

Figure S3. Comparison of states with the most municipal inclusionary zoning programs between National Zoning and Land Use Database and National Longitudinal Land Use Survey 2019 samples

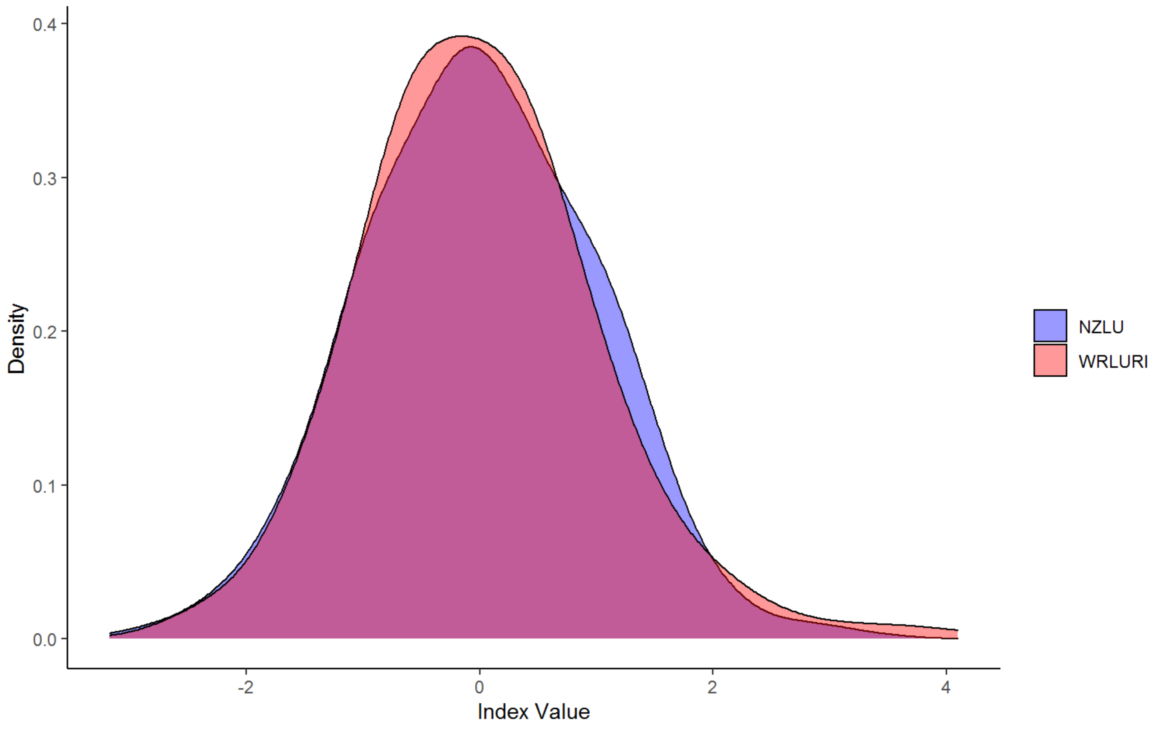

Figure S4. Density plots of ZRI (National Zoning and Land Use Database) and WRLURI (Wharton Residential Land Use Regulatory Index 2018) indices among all municipalities

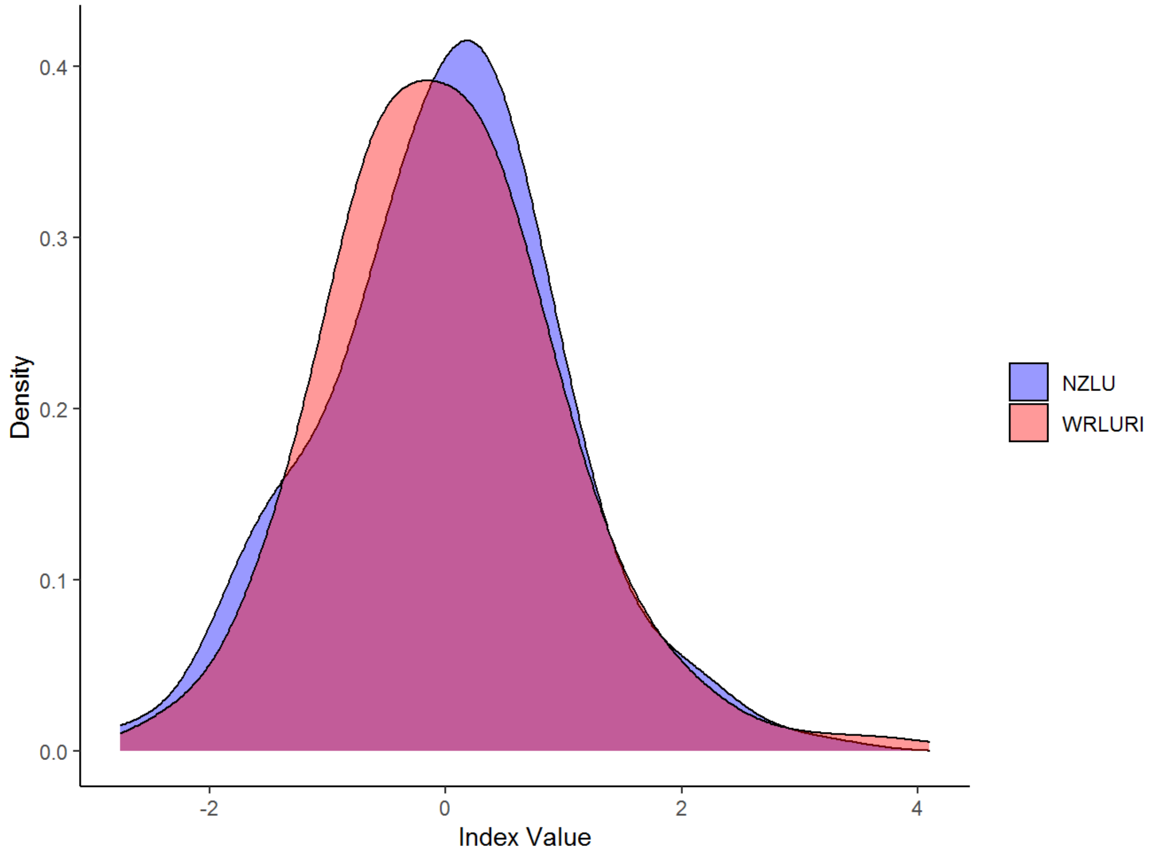

Figure S5. Density plots of ZRI (National Zoning and Land Use Database) and WRLURI (Wharton Residential Land Use Regulatory Index 2018) indices among all MSAs

References for Online Supplemental Material

Gyourko J, Saiz A and Summers A (2008) A New Measure of the Local Regulatory Environment for Housing Markets: The Wharton Residential Land Use Regulatory Index. *Urban Studies* 45(3): 693–729.

Gyourko J, Hartley JS and Krimmel J (2021) The local residential land use regulatory environment across U.S. housing markets: Evidence from a new Wharton index. *Journal of Urban Economics* 124: 103337.

Hlavac M (2018) *Stargazer: Well-Formatted Regression and Summary Statistics Tables*. Available at: https://CRAN.R-project.org/package=stargazer (accessed 15 June 2020).

Holloway SR, Wright R and Ellis M (2012) The Racially Fragmented City? Neighborhood Racial Segregation and Diversity Jointly Considered. *The Professional Geographer* 64(1): 63–82.

Social Explorer (2019) Population Density (Per Sq. Mile), Available at: https://www.socialexplorer.com/explore-tables (accessed 12 January 2022).

Reardon SF, Bischoff K, Owens A, et al. (2018) Has Income Segregation Really Increased? Bias and Bias Correction in Sample-Based Segregation Estimates. *Demography* 55(6): 2129–2160.

Roberto E (2016) The Divergence Index: A Decomposable Measure of Segregation and Inequality. arXiv:1508.01167 [stat.ME]. Available at: http://arxiv.org/abs/1508.01167 (accessed 16 July 2021).
